# Schlafen 11 Is Overexpressed in Multiple Myeloma and Undergoes Nucleolar Translocation in Response to Bortezomib

**DOI:** 10.1158/2767-9764.CRC-26-0162

**Published:** 2026-07-27

**Authors:** Yasuhiro Arakawa, Daiki Taniyama, Kazuhito Suzuki, Shingo Yano, Yves Pommier

**Affiliations:** 1 Laboratory of Molecular Pharmacology and Developmental Therapeutics Branch, Center for Cancer Research, NCI, NIH, Bethesda, Maryland.; 2 Division of Clinical Oncology and Hematology, Department of Internal Medicine, The Jikei University School of Medicine, Tokyo, Japan.; 3 Department of Clinical Pharmacology and Therapeutics, The Jikei University School of Medicine, Tokyo, Japan.

## Abstract

Proteins belonging to the Schlafen family are interferon-inducible and participate in the regulation of antiviral responses, immune signaling, and proteotoxic stress. Schlafen 11 (SLFN11) also kills cells with replicative damage, serving as a predictive biomarker for chemotherapeutic response. In this study, we examined SLFN11 expression and significance in multiple myeloma. The Cancer Genome Atlas and MMRF CoMMpass datasets were analyzed for *SLFN11* expression. Bone marrow and cell lines samples were analyzed for SLFN11 protein. SLFN11-knockout multiple myeloma cell lines were used to explore how SLFN11 affects bortezomib (BTZ) response. Retrospective analysis of the HOVON-65/GMMG-HD4 phase III trial (*n* = 327) assessed clinical relevance. *SLFN11* is consistently highly expressed across multiple myeloma subtypes (except CD1 and MAF/MAFB) and in normal plasma cells, and its expression strongly correlates with super-enhancer–driven plasma cell transcriptional programs. CD138-positive normal and myeloma plasma cells retain *SLFN11* expression even when proliferative activity (*MKI67*/Ki-67) increases with disease progression. BTZ, a first-line multiple myeloma treatment, induces SLFN11 nucleolar translocation with suppression of ribosomal RNA (rRNA) synthesis. Knocking out SLFN11 in multiple myeloma cells enhances BTZ sensitivity and exatecan resistance, supporting SLFN11’s protective role in proteotoxic stress and sensitizing role in replication stress. In the HOVON-65/GMMG-HD4 trial, *SLFN11*-low patients seemed to preferentially benefit from BTZ-based therapy, suggesting that *SLFN11* expression may guide therapeutic stratification in multiple myeloma.

**Significance:**

SLFN11 is highly expressed in normal and malignant plasma cells. BTZ induces SLFN11 nucleolar translocation, suppressing rRNA synthesis. SLFN11 confers BTZ resistance while sensitizing to topoisomerase I inhibitors. Low SLFN11 expression may identify patients more likely to benefit from BTZ-based therapy, providing a mechanistic rationale for SLFN11-guided therapeutic stratification.

## Introduction

Multiple myeloma arises from the clonal malignant proliferation of plasma cells (1). Multiple myeloma molecular subtypes are based on genetic alterations, including *MAF*/*MAFB* translocations (MF subtype), *CCND1*/*CCND3* translocations (CD1/CD2 subtypes), MMSET translocations (MS subtype), hyperdiploidy (HY subtype), and proliferation signatures (PR subtype; ref. 2). Comprehensive molecular profiling has expanded molecular understanding, identified high-risk genetic subtypes, and elucidated molecular underpinnings of multiple myeloma (3, 4). Despite advances in treatment modalities based on proteasome inhibitors, immunomodulatory drugs, and targeted therapies, precision medicine approaches remain limited, highlighting the need to identify additional biomarkers and molecular targets (1, 5).

**Figure 1.**
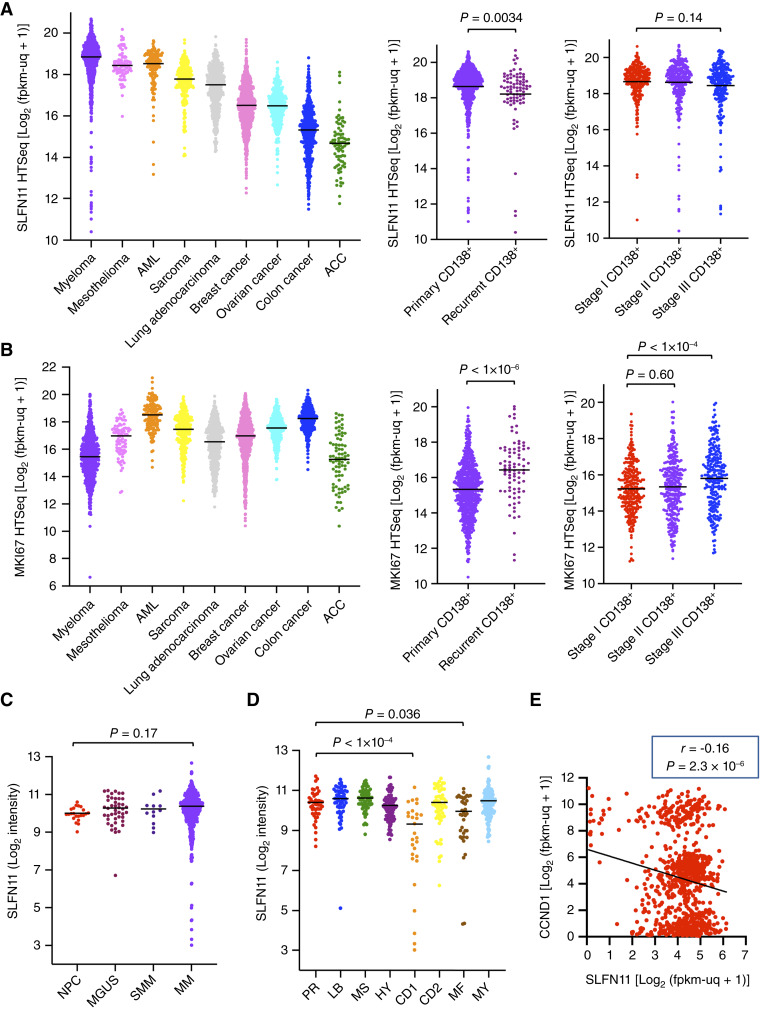
*SLFN11* and *MKI67* expression across cancer types and clinical parameters in multiple myeloma. **A,** Left, *SLFN11* expression levels [log_2_(fpkm-uq + 1)] across various cancer types from the TCGA and MMRF datasets, with multiple myeloma (MM) showing the highest expression. Middle, *SLFN11* expression in primary (newly diagnosed, *n* = 764) vs. recurrent (*n* = 80) bone marrow–derived CD138^+^ MM cells (*P* = 0.0034). Right, *SLFN11* expression across disease stages using ISS/R-ISS criteria (*P* = 0.14). **B,** Left, *MKI67* expression across cancer types, showing relatively low expression in MM. Middle, *MKI67* expression in primary vs. recurrent CD138^+^ cells (*P* < 1 × 10^−6^). Right, *MKI67* expression across disease stages, with stage III significantly higher than stage I (*P* < 1 × 10^−4^). **C,***SLFN11* expression (log_2_ intensity of probe 226743) across plasma cell disorders: NPCs (*n* = 22), MGUS (*n* = 44), SMM, (*n* = 12), and MM (*n* = 559; *P* = 0.17). This dataset (GSE5900/GSE2658) includes normal controls, which are not included in MMRF. **D,***SLFN11* expression across MM molecular subtypes (*n* = 559). CD1 and MF subtypes show significantly lower expression compared with the PR subtype (*P* < 1 × 10^−4^ and *P* = 0.036, respectively). **E,** Correlation between *SLFN11* and *CCND1* expression (r = −0.16, *P* = 2.3 × 10^−6^). Samples with low *SLFN11* expression [log_2_(fpkm-uq + 1) < 2] predominantly exhibit high *CCND1* expression. In all panels, bars represent mean values. ACC, adrenocortical carcinoma.

The Schlafen (SLFN) family comprises 10 genes in mice (Slfn1–10) and six in humans (6). The human genes (*SLFN* 5, *11*, *12*, *12L*, *13*, and *14*) are clustered on chromosome 17q12. In addition to the ribonuclease activity shared by all the Schlafen genes, SLFN11 binds to single-stranded DNA comprising immunostimulant CpG dinucleotides and to the single-stranded protein RPA. SLFN11 acts by irreversibly blocking replication and killing cancer cells in response to DNA damage produced by treatments that create replication stress (7). Thus, SLFN11 is a cell killing mediator in response to replicative DNA damage and an established predictive biomarker for multiple DNA-damaging agents across cancer types (7). Recently, SLFN11 has also been shown to kill cells by inducing TP53-independent apoptosis and blocking ribosome biogenesis (8, 9). The predictive value of *SLFN11* expression has been demonstrated in cell line models and across malignancies in which high *SLFN11* expression correlates with responses to chemotherapies targeting DNA replication (10–17). Epigenetic inactivation of *SLFN11* occurs in approximately 50% of cancer cell lines and in a large fraction of patient tumors, which leads to chemoresistance (7, 12). By contrast, some tumors consistently expressed high *SLFN11* such as acute myeloblastic leukemia (18), mesothelioma (19), and Ewing sarcoma (Fig. 1A; ref. 20).

SLFN11 also act as a regulator of protein translation (8, 9), a viral restriction factor (21), and an attenuator of endoplasmic reticulum and proteotoxic stress (22). These functions could have particular significance in malignancies such as multiple myeloma, which are characterized by high immunoglobulin production and can be prone to inherent proteotoxic stress, which explains the efficacy of proteasome inhibitors as cornerstone therapy of multiple myeloma.

Because the significance of SLFN11 in multiple myeloma has until now remained unknown, the present study aimed to characterize *SLFN11* expression across multiple myeloma subtypes, elucidate SLFN11 molecular interactions and pathway associations, and determine the impact of proteasome inhibition on *SLFN11* expression in multiple myeloma.

## Materials and Methods

### Cell lines and culture conditions

KMS-27 (RRID: CVCL_2993) and KMS-34 (RRID: CVCL_2996) multiple myeloma cell lines (23) were obtained directly from the Japanese Collection of Research Bioresources Cell Bank and maintained in RPMI-1640 medium (Gibco #11875093) supplemented with 10% fetal bovine serum (FBS; Gibco #A5256701), penicillin/streptomycin, and 2.5 μg/mL amphotericin B (FUJIFILM #019-23891). MM.1S multiple myeloma cell line (RRID: CVCL_8792) and U2OS osteosarcoma cell line (RRID: CVCL_0042) were obtained directly from the American Type Culture Collection. These cell lines were used within 6 months of receipt. MM.1S cells were cultured in the same medium as KMS cells. U2OS cells were maintained in McCoy’s 5a medium (Gibco #16600082) supplemented with 10% FBS and penicillin/streptomycin. Cell line identity was authenticated by the respective cell banks. All cell lines were tested for *Mycoplasma* contamination using the MycoAlert Mycoplasma Detection Kit (Lonza #LT07-318), most recently in December 2025, and were maintained at 37°C in a humidified atmosphere with 5% CO_2_.

### Patient specimens

Bone marrow aspirates and biopsies from patients with multiple myeloma were obtained with approval from the Ethics Committee of the Jikei University School of Medicine (approval number 37-008). Inclusion criteria: Patients diagnosed with multiple myeloma according to the International Myeloma Working Group criteria with adequate bone marrow specimens for analysis. Exclusion criteria: Patients with concurrent other hematologic malignancies or insufficient sample quality for immunohistochemical analysis. Written informed consent was obtained from patients where possible. For deceased patients or those lost to follow-up, the requirement for written consent was waived under an opt-out procedure approved by the Institutional Review Board (Ethics Committee of the Jikei University School of Medicine, approval number 37-008). The study was conducted in accordance with the Declaration of Helsinki. Normal bone marrow tissue arrays (BN29011a and BM241b) were purchased from TissueArray.Com. Sections of 2 to 4 μm thickness were prepared from formalin-fixed, paraffin-embedded (FFPE) specimens for immunohistochemical analysis. Sequential bone marrow samples were collected from patients at initial diagnosis and during disease progression or relapse following treatment. Normal bone marrow tissue arrays were used as controls.

### Dual immunohistochemical staining for CD138 and Ki-67

To evaluate the expression patterns of SLFN11 and CD138 in patient bone marrow samples, dual immunohistochemical staining was performed. Deparaffinized FFPE sections underwent antigen retrieval in high-pH buffer (DAKO #K8004) at 95°C for 20 minutes. After blocking endogenous peroxidase activity with 0.3% H_2_O_2_, sections were incubated with anti-SLFN11 antibody (Santa Cruz, #sc-515071, RRID: AB_3097730, 1:100 dilution) at room temperature for 60 minutes, followed by visualization using a polymer detection system (Histofine Simple Stain MAX-PO, Nichirei Biosciences #414341) and DAB chromogen (brown). Subsequently, sections were incubated with anti-CD138 antibody (Proteintech, 10593-1-AP, RRID: AB_2182736, 1:1,000 dilution) at 4°C overnight, followed by goat anti-rabbit IgG antibody, VECTASTAIN ABC-AP Kit (Vector Laboratories #AK-5000), and Vector Blue chromogen (blue). Sections were counterstained with nuclear fast red, dehydrated, and mounted for microscopic analysis. Stained slides were examined to quantify the proportions of CD138^+^/SLFN11+, CD138^+^/SLFN11−, CD138^−^/SLFN11+, and CD138^−^/SLFN11− cell populations. Cell populations were quantified by counting 1,000 cells per sample (including both CD138-positive and CD138-negative cells), except for samples with low cell density in which 500 cells were counted. For normal bone marrow samples, 200 CD138-positive cells were counted per sample.

For CD138/Ki-67 dual staining to assess proliferative activity, the same protocol was used with anti-Ki-67 antibody (Santa Cruz, #sc-101861, 1:100 dilution) replacing anti-SLFN11 antibody in the first staining step. The plasma cell proliferation index (PCPI) was calculated as the percentage of CD138-positive cells coexpressing Ki-67, using the same cell counting criteria described above.

### Immunofluorescence analysis with preextraction

For multiple myeloma cell lines, cells were applied to Superfrost Plus microscope slides (Electron Microscopy Sciences, 71869-10) using a Cytospin at 800 rpm for 4 minutes. To detect the chromatin-bound proteins (For Fig. 5D and E), the attached cells were briefly pretreated with ice-cold 0.1% Triton X-100 in PBS for 1 minute, followed by fixation in 4% paraformaldehyde (PFA) in PBS for 10 minutes at room temperature (9). For U2OS cell line, cells (5 × 10^4^ cells) were seeded on 8-well chamber slides (Nunc Lab-Tek II CC2, Thermo Fisher Scientific, #154941) except for high-throughput imaging experiments, in which 2,000 cells were seeded per well in 384-well plate (Perkin Elmer CellCarrier 384 plates, #6057300). Cells were treated with drugs as described in the figure legends. Cells were then preextracted in 0.1% Triton X-100 cytoskeleton buffer [10 mmol/L Tris (pH 6.8), 100 mmol/L NaCl, 300 mmol/L sucrose, MgCl_2_, 1 mmol/L ethylene glycol-bis(β-aminoethyl ether)-N,N,N′,N′-tetraacetic acid, 1 mmol/L ethylenediaminetetraacetic acid (EDTA), and 0.1% Triton X-100] for 5 minutes on ice, following fixation in 4% PFA for 15 minutes (24). For Fig. 6A and C, the attached cells were fixed with 4% PFA for 10 minutes, followed by permeabilization with 0.1% Triton X-100/PBS for 15 minutes. After fixation, cells were blocked with 4% bovine serum albumin (BSA) in PBS-T for 10 minutes and then incubated overnight at 4°C in a humidified chamber with primary antibodies diluted 1:1,000 in 4% BSA/PBS-T. The primary antibodies used were anti-SLFN11 (Santa Cruz, #sc-515071, RRID: AB_3097730), anti-nucleolin (Abcam, #ab129200, RRID: AB_11144140), and anti-UBF1 (Abcam, #ab244287). Following PBS-T washes, cells were incubated with Alexa Fluor 488–conjugated goat anti-mouse IgG or Alexa Fluor 568–conjugated goat anti-rabbit IgG (both 1:1,000 in 5% BSA/PBS-T) for 2 hours at room temperature. After additional PBS-T washes, the cells were mounted using Vectashield containing 4′,6-diamidino-2-phenylindole (DAPI) (VECTOR, H-1200). Imaging was performed using a Nikon SoRa spinning-disk confocal microscope equipped with a 60× objective lens. For the experiments in Fig. 5F, super-resolution imaging was performed, and post-acquisition deconvolution was applied exclusively to these images using NIS-Elements software (Nikon) to further enhance resolution and contrast.

### Immunofluorescence microscopy image analysis

Fiji (ImageJ, NIH; RRID: SCR_002285) software was used to quantify signal intensities at the single-cell level. Nuclear regions were identified by DAPI staining and used to define regions of interest across isogenic cell lines for the extraction of mean signal intensities. These values were used to generate individual plots. The processed data were subsequently imported into GraphPad Prism 11 (GraphPad Software; RRID: SCR_002798) for graphical representation.

### Automated image acquisition and image analysis

Stained 384-well plates were imaged on a Yokogawa CV7000S high-throughput spinning-disk confocal microscope using a 40× air objective (NA 0.95). DAPI and chromatin-bound SLFN11 were detected with 405 and 561 nm excitation, respectively. Single-plane images were acquired sequentially with a 16-bit sCMOS camera (2 × 2 binning; pixel size, 0.325 μm) using appropriate bandpass filters. Background and illumination corrections were performed using Yokogawa software, and images were exported as TIFF files. Image analysis was performed in Columbus 2.7.1 (PerkinElmer). Nuclei were segmented based on DAPI signal, and nuclear SLFN11 fluorescence intensity was quantified after treatment with camptothecin (CPT) or bortezomib (BTZ) for 4 hours. Nuclei with roundness <0.775 or touching the image border were excluded. Mean nuclear SLFN11 intensity was calculated per well and exported as text files for downstream analysis.

### Western blotting

For protein expression analysis, cells were lysed in RIPA buffer (50 mmol/L Tris-HCl pH 7.4, 150 mmol/L NaCl, 1% NP-40, 0.5% sodium deoxycholate, 0.1% SDS, and 1 mmol/L EDTA) supplemented with protease inhibitor cocktail (Roche #11836170001). Protein concentrations were determined using the Bio-Rad Protein Assay Kit (Bio-Rad #5000001). Equal amounts of protein (10 μg) were resolved by 5% to 20% SDS-PAGE and transferred to polyvinylidene difluoride membranes (Millipore #IPVH00005). After blocking with 5% nonfat dry milk in TBS-T, membranes were incubated with primary antibodies against SLFN11 (Santa Cruz #sc-515071, RRID: AB_3097730, 1:1,000) or β-actin (Sigma-Aldrich #A5441, RRID: AB_476744, 1:5,000) overnight at 4°C. Following incubation with horseradish peroxidase–conjugated secondary antibodies (Santa Cruz #sc-2357, #sc-2004), immunoreactive bands were visualized using ECL detection reagent (Atto #WSE-7110) and analyzed using Fiji software (NIH).

### Treatment with super-enhancer inhibitors

Multiple myeloma cell lines were treated with JQ1 (BET inhibitor, Sigma-Aldrich #SML1524-5MG) or THZ1·2HCl (CDK7 inhibitor, Selleck #S7549) to evaluate their effects on SLFN11, ZBTB38, and IRF2 protein expression. Stock solutions were prepared in DMSO. KMS-34 cells were treated with JQ1 (500 nmol/L) or THZ1 (200 nmol/L), and KMS-27 cells were treated with JQ1 (1 μmol/L) or THZ1 (50 nmol/L) for 0, 12, 24, and 48 hours. Cells were harvested at each time point for Western blot analysis as described above.

### CRISPR–Cas9-mediated SLFN11 knockout

SLFN11 knockout (KO) clones were generated in KMS-34 cells using the CRISPR–Cas9 system. SLFN11-targeting CRISPR constructs were kindly provided by Dr. Junko Murai (12) and modified according to the published protocol with minor adaptations. Briefly, KMS-27and KMS-34 cells were transfected with the CRISPR constructs using Nucleofector II Device with Nucleofector Kit V (Amaxa #VCA-1003) and program X-005 (25). Following transfection, cells were cultured in drug-free medium for 48 hours and subsequently selected with 0.25 μg/mL puromycin (Thermo Fisher Scientific, #A1113803) for 1 week. Single-cell clones were isolated by limiting dilution, expanded, and screened for SLFN11 KO by Western blotting and immunofluorescence analysis.

### Cell viability assays

Cell viability was assessed using either manual cell counting with trypan blue exclusion or ATPlight Luminescence Assay (Thermo Fisher Scientific #50-904-9890). For time-course experiments with BTZ (Fig. 7A), wild-type (WT) and SLFN11 KO cells were seeded in 24-well plates at a density of 2 × 10^5^ cells/mL and treated with indicated concentrations of BTZ. At 0, 24, 48, 72, and 96 hours, viable cells were counted after trypan blue staining.

For dose–response experiments (Fig. 7B–D), WT and SLFN11 KO cells were seeded in 96-well plates according to ATPlight protocol recommendations and treated with various concentrations of BTZ (0–15 nmol/L), CPT (Sigma-Aldrich #390238, 0–500 nmol/L), or exatecan (Sigma-Aldrich #SML3519, 0–15 nmol/L for KMS-34 or 0–50 nmol/L for KMS-27) for 72 hours. Cell viability was measured using ATPlight reagent according to the manufacturer’s instructions, and luminescence was recorded using a plate reader. All experiments were performed in triplicate. Data were normalized to untreated control cells (100% viability).

### Doxycycline-inducible SLFN11 expression system

U2OS cells stably expressing SLFN11 under the control of a doxycycline (DOX)-inducible promoter were generated via lentiviral transduction using the pLVX-TetOne-Blasticidin-3XFlag-SLFN11 construct. Forty-eight hours after infection, transduced cells were selected with 10 μg/mL blasticidin (Gibco). Cells were treated with DOX (Thermo Fisher Scientific, 5 μg/mL) for 72 hours to induce *SLFN11*.

### Ribosomal RNA synthesis assay

Followed by treating cells with drugs as described in the figure legends, cells were incubated with 5-ethynyl uridine (EU) for 1 hour (5 mmol/L for KMS-34 and KMS-27 cells; 1 mmol/L for U2OS cells), and nascent RNA was detected using the Click-iT RNA Alexa Fluor 488 Imaging Kit (Thermo Fisher Scientific, C10329), following the manufacturer’s instructions. EU fluorescence signal intensities were quantified using Fiji. All data were visualized using GraphPad Prism 11 (GraphPad Software).

### Protein synthesis assay

To evaluate global protein synthesis, the Click-iT HPG Alexa Fluor 488 Protein Synthesis Assay Kit (Thermo Fisher Scientific, #C10428) was used according to the manufacturer’s instructions. Cells were treated with 100 nmol/L BTZ for 2 or 4 hours. To label nascent proteins without inducing amino acid starvation, 50 μmol/L HPG was added directly to the culture medium during the final hour of BTZ treatment. After fixation and permeabilization, the click reaction was performed to detect incorporated HPG. Fluorescence signals were analyzed using a confocal microscope. HPG fluorescence signal intensities were quantified using Fiji. All data were visualized using GraphPad Prism 11.

### Analysis of public gene expression datasets

Public gene expression datasets were analyzed to investigate *SLFN11* expression across cancer types and multiple myeloma subtypes. The MMRF CoMMpass dataset (initial and relapsed bone marrow samples, *N* = 844) and The Cancer Genome Atlas (TCGA) Pan-Cancer dataset were analyzed and visualized using UCSC Xena browser (https://xenabrowser.net, data accessed February 2025; RRID: SCR_018938; refs. 26, 27). Preprocessed RNA sequencing (RNA-seq) data with STAR aligner (RRID: SCR_004463) mapping and FPKM-UQ normalization were downloaded and analyzed. Microarray data of normal plasma cells (NPC) and sequential stages of myeloma progression were downloaded from the Gene Expression Omnibus (GEO; RRID: SCR_005012) database. Specifically, we utilized datasets GSE5900 and GSE2658 for analyzing *SLFN11* expression across NPCs, monoclonal gammopathy of undetermined significance (MGUS), smoldering multiple myeloma (SMM), and symptomatic multiple myeloma ( refs. 2, 28). Correlation analysis between drug sensitivity and *SLFN11* expression from the Cancer Therapeutics Response Portal database was performed and visualized using CellMiner CDB (https://discover.nci.nih.gov/rsconnect/cellminercdb/; ref. 29). Gene expression values were normalized as log_2_(fpkm-uq + 1) for RNA-seq data analysis.

### Retrospective clinical subgroup analysis (HOVON-65/GMMG-HD4)

Gene expression data from the HOVON-65/GMMG-HD4 phase III trial were obtained from GEO (GSE19784; ref. 30). Event-free survival (EFS) data and treatment group assignments for 327 patients for whom gene expression profiling data were available [PAD (BTZ, doxorubicin, and dexamethasone) group: *n* = 169, VAD (vincristine, doxorubicin, and dexamethasone) group: *n* = 158] were obtained from the GESTURE repository (https://github.com/jubels/GESTURE; ref. 31). SLFN11 expression levels were quantified using probe 226743_at on the Affymetrix HG-U133 Plus 2.0 array (GPL570) and verified using the hgu133plus2.db Bioconductor annotation package in R. Patients were divided into *SLFN11* high-expression and *SLFN11* low-expression groups based on the cohort median (log_2_ = 8.12). Kaplan–Meier survival curves were constructed and compared using the log-rank test. Patients with unknown International Staging System (ISS) stage (*n* = 23) were excluded, and an ISS-adjusted Cox proportional hazards regression analysis was performed. The interaction between the SLFN11 group and the treatment group was evaluated using a Cox model that included an SLFN11 group × treatment group interaction term. All analyses were performed using the survival and survminer packages in R version 4.4.3.

### Pathway analysis and gene set enrichment analysis

Differential gene expression (DGE) analysis and gene set enrichment analysis (GSEA) were performed using the analytic functions available in Xena browser (27). Samples from the MMRF CoMMpass study, including both newly diagnosed and relapsed bone marrow specimens, were divided into SLFN11-high and SLFN11-low groups based on median SLFN11 expression. DGE analysis was conducted using Xena browser’s limma-voom implementation (32, 33).

Gene Ontology (GO) enrichment analysis was performed using Xena browser’s Enrichr integration to identify significantly enriched biological processes, molecular functions, and cellular components in differentially expressed genes between *SLFN11*-high and *SLFN11*-low samples (34, 35). GSEA was conducted using the blitzGSEA function within Xena browser with gene sets from the Molecular Signatures Database (MSigDB), including both hallmark and ontology gene sets (36). Positive normalized enrichment scores indicate enrichment in *SLFN11*-high samples, whereas negative scores indicate enrichment in *SLFN11*-low samples.

Heatmaps of differentially expressed genes were generated using Xena browser’s visualization tools to display expression patterns across samples, with particular focus on genes related to endoplasmic reticulum (ER) stress response, unfolded protein response (UPR), ribosomal proteins, and ubiquitin–proteasome system components.

### Statistical analyses

Statistical analyses were performed using GraphPad Prism 11 (GraphPad Software; RRID: SCR_002798). Comparisons between two groups were conducted using either unpaired *t* test or Mann–Whitney U test depending on data distribution. For multiple group comparisons, one-way ANOVA with Tukey’ *post hoc* test or Kruskal–Wallis test with Dunn *post hoc* test was applied as appropriate. Correlations between gene expression levels were evaluated using Pearson’s correlation coefficient. For all analyses, *P* values less than 0.05 were considered statistically significant. Details regarding data presentation are provided in the respective figure legends.

## Results

### *SLFN11* expression is consistently elevated in multiple myeloma

Analysis of TCGA and MMRF CoMMpass datasets (4, 37) revealed that multiple myeloma expresses high *SLFN11* transcript levels, exceeding mesothelioma, sarcoma, and acute myeloid leukemia [AML; Fig. 1A (left); refs. 16, 18–20]. Only 2.8% show low *SLFN11* expression [log_2_(fpkm-uq + 1) < 16; Fig. 1A].

Examination of different disease stages and conditions shows that *SLFN11* expression is statistically lower in recurrent bone marrow samples than in samples from primary disease [*P* = 0.0034; Fig. 1A (middle)]. However, the magnitude of this difference is relatively small, suggesting that *SLFN11* expression remains high despite disease recurrence. No significant variation in *SLFN11* expression was observed across different disease stages [*P* = 0.14; Fig. 1A (right)].

We examined *MKI67* expression, as both SLFN11 and proliferation status predict sensitivity to DNA-damaging agents (38, 39). Multiple myeloma typically exhibits relatively low proliferative activity, which may contribute to conventional chemotherapy resistance (40). *MKI67* expression was notably lower in multiple myeloma than other cancer types [Fig. 1B (left)]. Yet, *MKI67* was significantly elevated in recurrent versus primary samples (*P* < 1 × 10^−6^, >2-fold) and stage III versus stage I [*P* < 1 × 10^−4^; Fig. 1B (middle and right)].

To explore *SLFN11* expression during plasma cell disorder progression, we analyzed *SLFN11* levels in NPCs, MGUS, SMM, and symptomatic multiple myeloma using published gene expression microarray data (GSE5900 and GSE2658; ref. 28). No statistically significant difference was observed between these groups (*P* = 0.17; Fig. 1C), indicating that NPCs inherently express high levels of *SLFN11* and that this elevated expression is maintained throughout malignant transformation and progression to symptomatic multiple myeloma.

Analysis of 1,019 cancer cell lines in public databases (https://discover.nci.nih.gov/cellminercdb/) revealed that approximately one thirds of multiple myeloma cell lines exhibit *SLFN11* deficiency and nearly half show high *MKI67* expression (Supplementary Fig. S1A), contrasting with the consistently high *SLFN11* expression (97.2%) and low proliferative activity observed in patient-derived multiple myeloma samples. This difference is likely due to the fact that cell line selection favors clones with high proliferation potential.

### *SLFN11* expression across multiple myeloma molecular subtypes

We next explored whether *SLFN11* expression differs across the established multiple myeloma molecular subtypes. Analysis of the microarray dataset GSE2658 (2) revealed variation in *SLFN11* expression among the molecular subgroups (Fig. 1D). Both the CD1 and MAF/MAFB (MF) subtypes exhibit significantly lower *SLFN11* expression compared with the proliferation (PR) subtype (*P* < 1 × 10^−4^ and *P* = 0.036, respectively). Accordingly, samples with low *SLFN11* (log_2_ intensity < 7) were predominantly classified as CD1 subtype. No significant difference in *SLFN11* expression was observed between the PR subtype and other molecular subtypes: MMSET (MS), hyperdiploid (HY), low bone disease (LB), and CD2 (Fig. 1D).

As dysregulation of D-type cyclins is a known driver of multiple myeloma pathogenesis (2), we examined cyclin D expression in the MMRF CoMMpass dataset. Samples with low *SLFN11* expression [log_2_(fpkm-uq + 1) < 2] demonstrated high expression of *CCND1* (Fig. 1E). Although not all *CCND1*-high samples showed low *SLFN11* expression, the inverse relationship suggests that *CCND1* overexpression is associated with *SLFN11* suppression in a distinct subset of multiple myeloma.

Additional analysis of the MMRF CoMMpass dataset across the recently defined RNA expression subtypes revealed that a subset of samples in the CD subtypes (CD1, CD2a, and CD2b) exhibit low *SLFN11* expression. We also observed notable differences in *SLFN11* expression among hyperdiploid (HRD) samples, varying by their specific additional genetic alterations (Supplementary Fig. S1B). Yet, copy number–based subtypes showed minimal variation in *SLFN11* expression (Supplementary Fig. S1C), suggesting that high *SLFN11* expression in multiple myeloma is primarily driven by transcriptional upregulation.

### CD138-positive myeloma cells express SLFN11 in patient bone marrow samples

**Figure 2.**
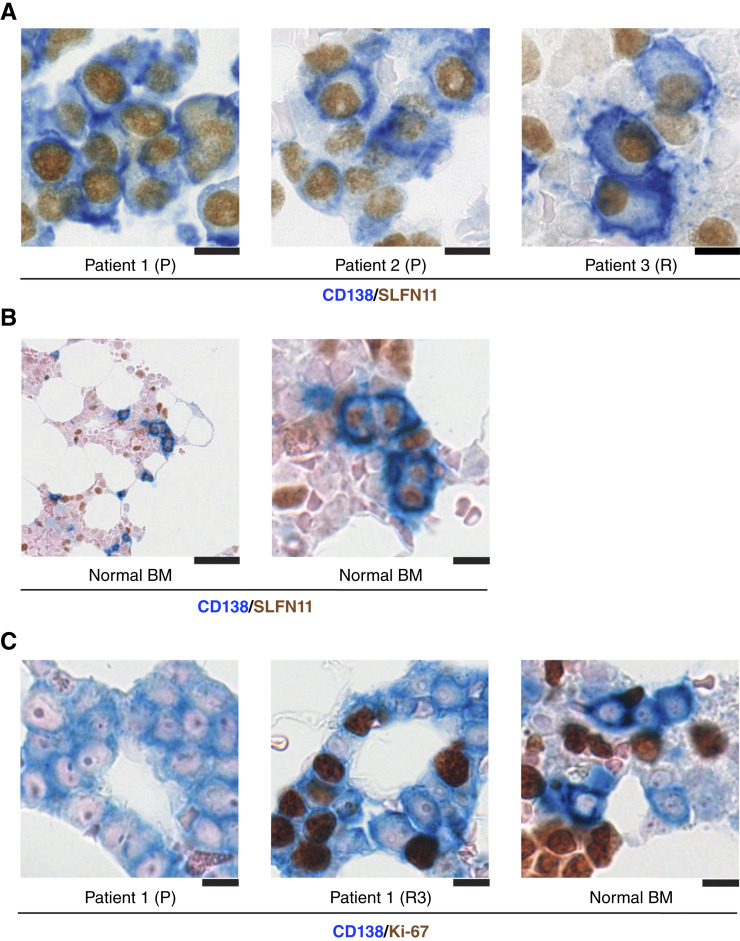
Expression of SLFN11, CD138, and Ki-67 in multiple myeloma (MM) patient samples and normal bone marrow. **A,** Representative dual immunohistochemical staining for CD138 (blue, membrane) and SLFN11 (brown, nuclear) in bone marrow samples from patients with MM. Patients 1 and 2 are primary (P; newly diagnosed), whereas patient 3 (R) represents a sample from a recurrent patient. 81%–98% CD138-positive myeloma cells express SLFN11. **B,** Dual immunohistochemical staining for CD138 (blue) and SLFN11 (brown) in normal bone marrow. Left, low magnification. Right, high magnification of the same field. Normal bone marrow contains 5.5%–6.8% plasma cells, of which 72%–85% demonstrate SLFN11 positivity, indicating that *SLFN11* expression is an intrinsic feature of NPCs. Scale bars, 50 μm (left) and 10 μm (right). **C,** Representative dual immunohistochemical staining for CD138 (blue) and Ki-67 (brown, nuclear) to assess proliferative activity. Left and middle, patient 1 at primary diagnosis (P) and third relapse (R3), respectively. Right, normal bone marrow. The PCPI, defined as the percentage of CD138-positive cells coexpressing Ki-67, is markedly elevated in myeloma specimens compared with normal bone marrow, with further increases observed at relapse. Scale bars, 10 μm. BM, bone marrow.

To determine SLFN11 protein expression in multiple myeloma patient bone marrow samples, we performed double immunocytochemical staining of SLFN11 and CD138, an established marker routinely used to identify malignant cells in multiple myeloma. Figure 2A shows that CD138-positive myeloma cells (81%–98%) coexpress SLFN11. Staining patterns, both of primary (P) and relapsed (R) specimens, revealed that SLFN11 (brown) is mainly localized to the nucleus, whereas CD138 (blue) shows characteristic membrane staining.

Examination of normal bone marrow samples containing plasma cells, representing 5% to 7% of total bone marrow cells, demonstrated that NPCs are generally SLFN11 positive (72%–85%; Fig. 2B). These findings demonstrate that SLFN11 expression is an intrinsic feature of both normal and malignant plasma cells.

To assess single-cell proliferative activity, we performed CD138/Ki-67 double staining and calculated the PCPI, defined as the percentage of CD138-positive cells coexpressing Ki-67 (41). Consistent with our transcriptome findings (Fig. 1B), comparison of multiple myeloma patient samples with normal bone marrow demonstrated elevated PCPI in myeloma specimens, with further increase observed in relapsed samples (Fig. 2C).

Quantitative analysis of four distinct cell populations based on SLFN11/CD138 expression patterns (CD138^+^/SLFN11+, CD138^+^/SLFN11−, CD138^−^/SLFN11+, and CD138^−^/SLFN11−) was performed on sequential bone marrow samples from three patients with multiple myeloma and normal bone marrow controls (Supplementary Fig. S2A and S2B). Although the total percentage of CD138-positive myeloma cells fluctuated over time with disease progression and treatment response, most myeloma cells consistently maintained SLFN11 expression throughout the disease course. The PCPI analysis confirmed progressive increases with each relapse across all three patients, although the absolute values remained relatively modest in patients 2 and 3 compared with patient 1. In contrast, normal bone marrow samples showed minimal PCPI values (Supplementary Fig. S2C).

Taken together, these observations establish SLFN11 expression as a feature of CD138-positive myeloma cells, which is maintained from NPCs through malignant transformation and multiple myeloma disease progression.

### SLFN11 is expressed with plasma cell differentiation markers

**Figure 3.**
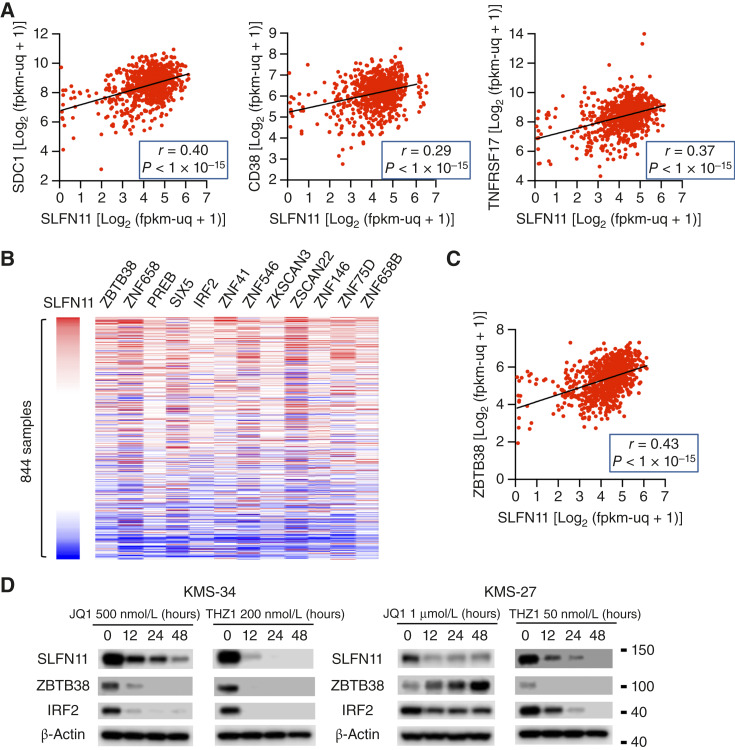
Correlations between the expression of *SLFN11* and plasma cell markers and *SLFN11* transcriptional regulation in multiple myeloma (MM). **A,** Scatter plots demonstrating positive correlations between *SLFN11* expression and plasma cell markers (*n* = 844 MM samples from the MMRF CoMMpass study. **B,** Heatmap displaying the mRNA expression of the top 12 transcription factors and epigenetic enzymes with the strongest correlation with *SLFN11* expression (samples are from the MMRF CoMMpass study). Red, high expression; blue, low expression. **C,** Scatter plot highlighting the positive correlation between *SLFN11* and *ZBTB38* expression. **D,** Representative Western blots showing time-dependent effects of super-enhancer inhibitors on SLFN11, ZBTB38, and IRF2 protein levels in MM cell lines. Left, KMS-34 cells treated with JQ1 (BET inhibitor, 500 nmol/L) or THZ1 (CDK7 inhibitor, 200 nmol/L) for 0, 12, 24, and 48 hours. Right, KMS-27 cells treated with JQ1 (1 μmol/L) or THZ1 (50 nmol/L) for the same time points. β-Actin serves as a loading control. TF, transcription factor.

To explore the relationship between SLFN11 and known plasma cell markers, we analyzed the MMRF CoMMpass dataset, in which clinical samples are enriched for plasma cells through CD138 antibody–based magnetic bead selection prior to RNA-seq. Significant positive correlations were observed between *SLFN11* expression and the expression of established plasma cell differentiation markers: *CD138* (*SDC1* HUGO name), *CD38*, and *BCMA* (TNF receptor superfamily member 17; *TNFRSF17*; Fig. 3A).

In contrast, *SLFN11* expression showed no significant correlation with markers of other hematopoietic lineages, including *CD8A* (T-cell marker), *CD79A* (B-cell marker), or *CD68* (monocyte/macrophage marker; Supplementary Fig. S3A). This specificity confirms that the high *SLFN11* expression observed in multiple myeloma is associated with the plasma cell differentiation program rather than contaminating immune cell populations.

### Transcriptional regulation of SLFN11 in multiple myeloma

To identify potential transcriptional regulators of *SLFN11* in multiple myeloma, we examined the correlations between *SLFN11* expression and the expression of 122 transcription factors and epigenetic enzymes involved in plasma cell differentiation (Supplementary Fig. S3B; ref. 42). The 12 factors showing strongest correlation with *SLFN11* are presented in Fig. 3B, with *ZBTB38* most highly correlated (r = 0.43; Fig. 3C). As the strongest correlate among the factors examined, *ZBTB38* is presented here as a representative member of the plasma cell transcriptional program; this correlation likely reflects coregulation within the plasma cell program rather than direct transcriptional control of *SLFN11* by *ZBTB38*.

As super-enhancers have been shown to regulate key transcription factors essential for multiple myeloma cell state, including *IRF4*, *PRDM1*, *MYC*, and *XBP1* (43), we treated two multiple myeloma cell lines (KMS-34 and KMS-27) with inhibitors of super-enhancer–mediated gene expression. Both JQ1 (BET inhibitor) and THZ1 (CDK7 inhibitor), which target super-enhancer–mediated transcriptional control (44), reduced SLFN11 protein levels in a time-dependent manner, along with concomitant decrease in IRF2 (Fig. 3D). Whereas the ZBTB38 response was cell line–dependent, the reduction of SLFN11 itself was consistent across both lines, indicating that *SLFN11* expression is under super-enhancer control in multiple myeloma.

By contrast, *FLI1* expression, a known transcriptional regulator of *SLFN11* in Ewing sarcoma (20) and leukemia (18), showed only a weak and nonsignificant correlation with *SLFN11* expression in multiple myeloma (r = 0.15; Supplementary Fig. S3C), implying context-specific regulatory mechanisms for *SLFN11* expression (20). These results establish a strong association between *SLFN11* expression and the plasma cell differentiation super-enhancer program in multiple myeloma.

### Molecular pathway signatures associated with *SLFN11* expression in multiple myeloma

**Figure 4.**
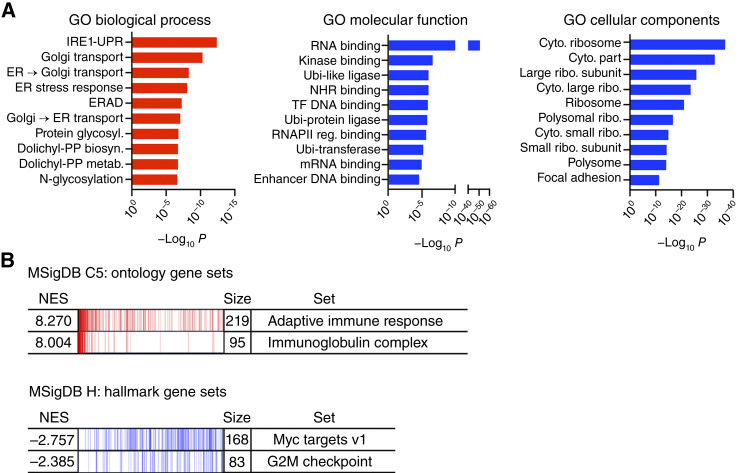
Pathway analysis of *SLFN11*-associated genes in multiple myeloma (MM). **A,** GO enrichment analysis comparing MM samples with the highest and lowest *SLFN11* expression (*n* = 844, divided by median expression). Red bars indicate pathways upregulated in *SLFN11*-high samples, whereas blue bars show pathways enriched in *SLFN11*-low samples. Left, biological processes; middle, molecular functions; right, cellular components. The *x*-axis represents −log_10_ (*P* value). **B,** GSEA comparing *SLFN11*-high vs. *SLFN11*-low samples. Top, MSigDB C5 ontology gene sets showing positive enrichment in *SLFN11*-high samples, including adaptive immune response (NES = 8.270) and immunoglobulin complex (NES = 8.004). Bottom, MSigDB hallmark gene sets showing negative enrichment in *SLFN11*-high samples, including MYC targets v1 (NES = −2.757) and G2M checkpoint (NES = −2.385). NES, normalized enrichment score.

To gain insights into the functional implications of *SLFN11* expression in multiple myeloma, we performed pathway analyses comparing the samples with the highest and lowest *SLFN11* expression. GO enrichment analysis and heatmap visualization of differentially expressed genes across 844 multiple myeloma samples reveal distinct biological processes associated with *SLFN11* expression in multiple myeloma (Fig. 4A). *SLFN11*-high samples showed significant enrichment in pathways related to ER stress, UPR, ER-associated degradation (ERAD), and Golgi transport mechanisms. These pathways are critical for plasma cells to manage their high immunoglobulin secretory burden. In contrast, GO analysis revealed that *SLFN11*-low samples preferentially express genes related to ubiquitin–protein ligase activity, ubiquitin transferase functions, and ribosomal protein transcription, suggesting interactions between SLFN11 and protein quality control mechanisms (8, 9).

GSEA provides additional insights into the molecular signatures associated with *SLFN11* expression (Fig. 4B). *SLFN11*-high samples show significant positive enrichment in adaptive immune response, immunoglobulin complex, and antigen binding gene sets. Conversely, samples with the lowest *SLFN11* expression exhibit enrichment in key proliferation-related pathways such as G2M checkpoint and MYC targets.

Heatmap visualization of the differentially expressed genes across 844 multiple myeloma samples illustrates the coordination between *SLFN11* expression and genes involved in ER stress response, ERAD, ubiquitin–protein ligase activity, ubiquitin transferase functions, and ribosomal components (Supplementary Fig. S4A). More comprehensive GSEA analysis reveals that *SLFN11*-low samples are enriched in pathways related to TNFα signaling via NFκB, inflammatory response, apoptosis, and proliferation markers, while samples with the highest *SLFN11* expression show positive enrichment in cytoplasmic translation, ribosome function, and lymphocyte-mediated immunity (Supplementary Fig. S4B).

Collectively, these findings suggest that *SLFN11* expression in multiple myeloma is associated with plasma cell phenotype characterized by high secretory activity and ER stress response, whereas *SLFN11*-low samples exhibit features of increased proliferation and inflammatory signaling.

### SLFN11 translocation to nucleoli upon BTZ treatment

**Figure 5.**
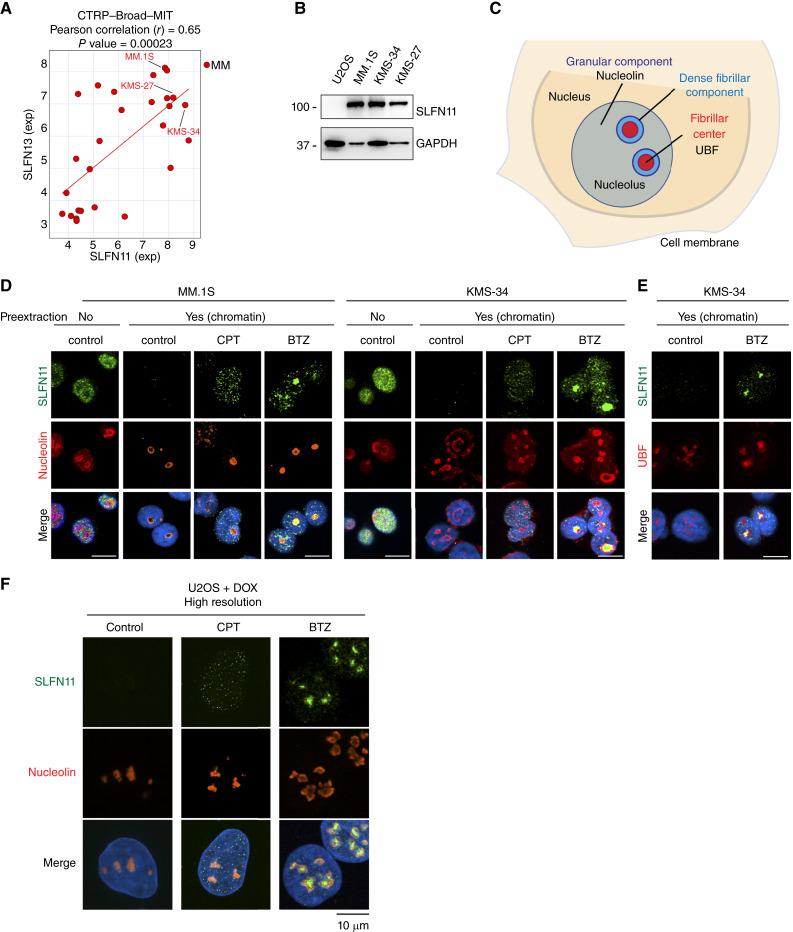
SLFN11 expression in multiple myeloma (MM) cell lines and SLFN11 translocation to nucleoli by BTZ. **A,** Correlation between the expression of *SLFN11* and *SLFN13* in the Cancer Therapeutics Response Portal (CTRP)–Broad–MIT cell line database (https://discover.nci.nih.gov/cellminercdb/). Correlation coefficient and *P* value are indicated above the figure. **B,** Representative SLFN11 immunoblots in MM.1S, KMS-34, and KMS-27 cell lines. Human osteosarcoma cell line U2OS was used as a negative control. **C,** Scheme of the nucleolus comprising the fibrillar center, the dense fibrillar component, and the granular component. **D,** Representative immunofluorescence images showing total SLFN11 (green, left, no preextraction) and chromatin-bound SLFN11 (green, right) with nucleolin (red) and DAPI (blue) in MM.1S and KMS-34 cells after 4-hour treatment with BTZ [0.5 μmol/L (MM.1S), 5 μmol/L (KMS-34)] or CPT [1 μmol/L (MM.1S), 5 μmol/L (KMS-34)]. Scale bars, 10 μm. **E,** Representative immunofluorescence images showing total chromatin-bound SLFN11 (green) with UBF (red) and DAPI (blue) in KMS-34 cells after 4-hour treatment. Scale bars, 10 μm. **F,** Representative immunofluorescence image showing BTZ-induced SLFN11 nucleolar translocation in U2OS with DOX-inducible SLFN11 expression. Cells were treated with CPT or BTZ (1 μmol/L) for 4 hours.

Because proteasome inhibitors are used as first-line therapy in multiple myeloma, we examined the cellular distribution of SLFN11 in multiple myeloma cell lines treated with the proteasome inhibitor BTZ. To select multiple myeloma cell lines with high SLFN11 expression, we used the CellMinerCDB website (https://discover.nci.nih.gov/cellminercdb/; ref. 45) to analyze the Broad Institute cell line genomic and drug response database (Fig. 5A). Immunoblot analysis confirmed SLFN11 protein expression in the multiple myeloma cell lines MM.1S, KMS-34, and KMS-27, whereas the osteosarcoma cell line U2OS, known to lack SLFN11 expression (46), served as a negative control (Fig. 5B).

Given the recent findings linking SLFN11 and nucleolar functions (9), we used established markers to identify the distinct nucleolar regions: upstream binding factor (UBF) for the fibrillar center and nucleolin for the granular component (Fig. 5C). Standard immunofluorescence of untreated MM.1S and KMS-34 cells showed the expected predominantly diffuse SLFN11 staining (green) in the nucleus. The SLFN11 staining of untreated cells was largely excluded from nucleoli in MM.1S cells, as evidenced by the minimal overlap with nucleolin (red), although variable nucleolar overlap was observed in KMS-34 cells [Fig. 5D (left)]. This pattern recapitulates the SLFN11 distribution observed in untreated bone marrow samples (see Fig. 2A).

As expected under baseline conditions, upon mild detergent preextraction, SLFN11 was no longer detectable due to its known loose chromatin binding (47). By contrast, following treatment with CPT, a topoisomerase I (TOP1) poison known to induce replicative DNA damage, SLFN11 chromatin foci were readily detectable throughout the nuclei without enrichment in nucleoli (Fig. 5D; Supplementary Fig. S5A; refs. 17, 47). Unexpectedly, treatment with BTZ only induced intense SLFN11 staining in nucleoli both in the MM.1S and KMS-34 cells, as evidenced by increased co-localization of SLFN11 with nucleolin (Fig. 5D; Supplementary Fig. S5A) and UBF (Fig. 5E). BTZ-induced SLFN11 nucleolar translocation was also observed with DOX-inducible SLFN11-expressing osteosarcoma U2OS cells (Fig. 5F; Supplementary Fig. S5B and S5C), implying that this effect is not limited to multiple myeloma cells.

### SLFN11 suppresses ribosomal RNA synthesis in response to BTZ

**Figure 6.**
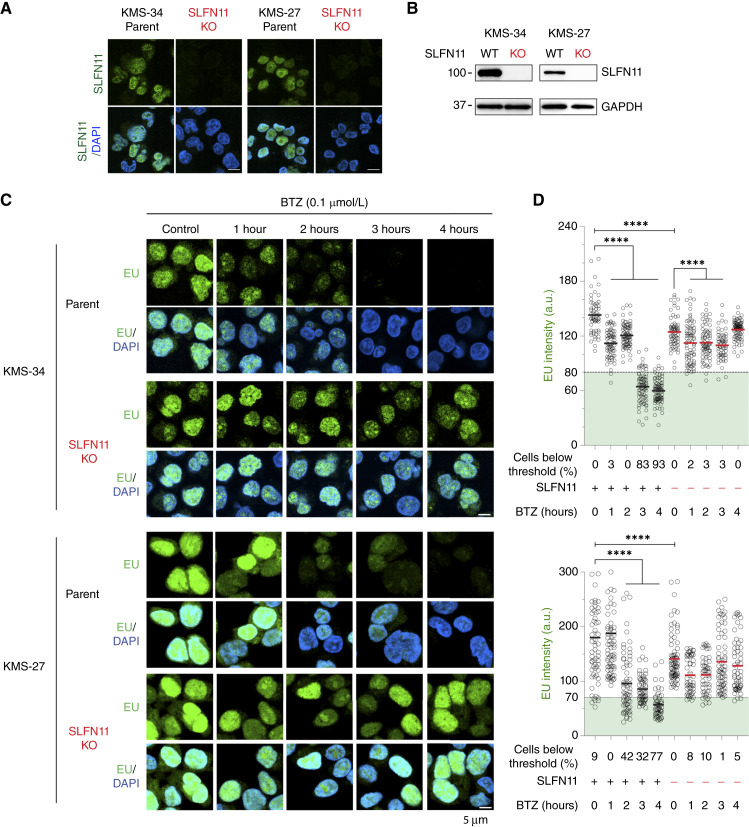
SLFN11-dependent suppression of rRNA synthesis by BTZ. **A,** Representative immunofluorescence images showing total SLFN11 (green, no preextraction) and DAPI (blue) in parental and SLFN11 KO KMS-34 cells. Scale bars, 10 μm. **B,** SLFN11 immunoblots for whole-cell lysates from WT and SLFN11 KO clones of KMS-34 cells. **C,** Representative immunofluorescence images showing EU incorporation (green) and DAPI (blue). Top, KMS-34 cells. Bottom, KMS-27 cells. WT and SLFN11 KO clones were treated with BTZ (0.1 μmol/L) for indicated time points. Scale bars, 5 μm. **D,** Quantification of EU signal intensities in individual cells. Mean ± SEM are shown. Top, KMS-34 cells (threshold = 80). Bottom, KMS-27 cells (threshold = 70). Percentages of cells below threshold are indicated. ****, *P* < 0.0001 (one-way ANOVA). a.u., arbitrary units.

Because the BTZ-induced tight binding of SLFN11 to the fibrillar center of nucleoli suggested a potential role for SLFN11 in regulating nucleolar functions in response to proteotoxic stress, we investigated the functional significance of the nucleolar translocation of SLFN11 in response to BTZ. To do so, we generated SLFN11 KO clones from the KMS-34 and KMS-27 cell lines using the CRISPR–Cas9 technology (12, 47). KO was confirmed by immunofluorescence and immunoblot analyses (Fig. 6A and B). To assess the impact of SLFN11 on ribosomal RNA (rRNA) synthesis (9), we performed EU incorporation assays (9). In untreated conditions, both WT and SLFN11 KO cells showed robust EU signals, reflecting active RNA synthesis. Following BTZ treatment, EU incorporation was significantly reduced in WT cells, whereas SLFN11 KO cells maintained high levels of EU incorporation (Fig. 6C). Quantitative analysis confirmed the significant difference in EU signals between WT and SLFN11 KO cells after BTZ treatment (*P* < 0.0001; Fig. 6D).

To further establish the role of SLFN11 in suppressing rRNA synthesis under proteotoxic stress, we used DOX-inducible SLFN11-expressing U2OS cells (Supplementary Fig. S6A). EU incorporation assays demonstrated that induction of *SLFN11* expression significantly suppresses rRNA synthesis following BTZ treatment compared with noninduced controls (Supplementary Fig. S6B and S6C). Additionally, to assess global translation after BTZ treatment, we performed an HPG assay. We observed marked reduction in HPG signal intensity following BTZ treatment in SLFN11 KMS-27 cells. This result indicates that SLFN11 nucleolar translocation suppresses global translation after BTZ treatment. Collectively, these findings demonstrate that BTZ induces the translocation of SLFN11 to nucleoli not only in multiple myeloma cell lines but also in osteosarcoma U2OS cells and that this translocation is associated with suppression of rRNA synthesis.

### SLFN11 multiple myeloma KO cells show enhanced sensitivity to BTZ

**Figure 7.**
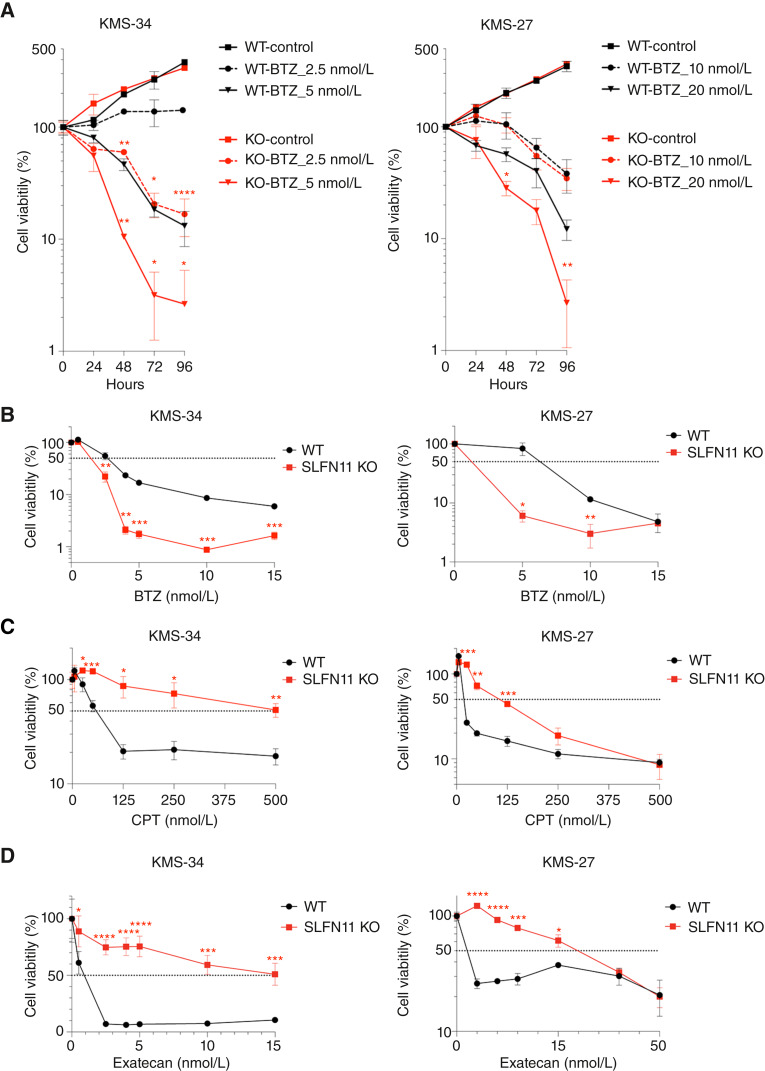
SLFN11 confers resistance to BTZ and sensitizes to TOP1 poisons. **A,** Time-course analysis of cell viability in WT and SLFN11 KO cells treated with BTZ. Left, KMS-34 cells treated with 2.5 or 5 nmol/L BTZ. Right, KMS-27 cells treated with 10 or 20 nmol/L BTZ. Cell viability was assessed by trypan blue exclusion at 0, 24, 48, 72, and 96 hours. **B–D,** Dose–response curves of WT and SLFN11 KO cells treated for 72 hours with BTZ (0–15 nmol/L; **B**), CPT (0–500 nmol/L; **C**) or exatecan (0–15 nmol/L for KMS-34, 0–50 nmol/L for KMS-27; **D**). Cell viability was measured using ATP assay. All experiments were performed in triplicate. Data are mean ± SD. *, *P* < 0.05; **, <0.01; ***, <0.001; ****, <0.0001.

To further explore the functional role of SLFN11 in multiple myeloma cells, we tested whether SLFN11 affects the sensitivity of multiple myeloma cells proteasome inhibitors. Cell growth experiments revealed that knocking out SLFN11 both for the KMS-34 and KMS-27 cell lines showed significantly enhanced sensitivity to BTZ (Fig. 7A). Dose–response experiments using the ATPlite assay confirmed the increased sensitivity to BTZ in SLFN11 KO cells (Fig. 7B). Conversely, and as expected (37), SLFN11 KO multiple myeloma cell lines showed resistance to CPT (Fig. 7C) and exatecan, the payload of many antibody–drug conjugates (ADC) in clinical trials and preclinical development (Fig. 7D). These results confirm SLFN11’s role as a determinant of sensitivity to DNA-damaging agents in multiple myeloma cells. They also demonstrate the resistance of multiple myeloma cells to BTZ in the absence of SLFN11 expression, consistent with a protective role for SLFN11 against proteotoxic stress (9, 22).

### SLFN11 expression is associated with BTZ activity in a clinical dataset

To explore the clinical relevance of our experimental findings, we searched published gene expression datasets from randomized clinical trials comparing first-line proteasome inhibitor–containing versus proteasome inhibitor–free therapies in multiple myeloma. The HOVON-65/GMMG-HD4 phase III trial (GSE19784) met these criteria, comparing PAD versus VAD induction, followed by high-dose melphalan with autologous stem cell transplantation and maintenance therapy (BTZ vs. thalidomide) in newly diagnosed multiple myeloma with pretreatment gene expression data available for 327 patients (PAD, *n* = 169; VAD, *n* = 158; refs. 30, 31). Both regimens share doxorubicin and dexamethasone, differing only in the substitution of vincristine with BTZ, providing a comparison for BTZ-specific effects, although a contribution of doxorubicin cannot be fully excluded. *SLFN11* expression was determined using probe 226743_at (Affymetrix HG-U133 Plus 2.0, GPL570), and patients were dichotomized at the cohort median (log_2_ = 8.12).

**Figure 8.**
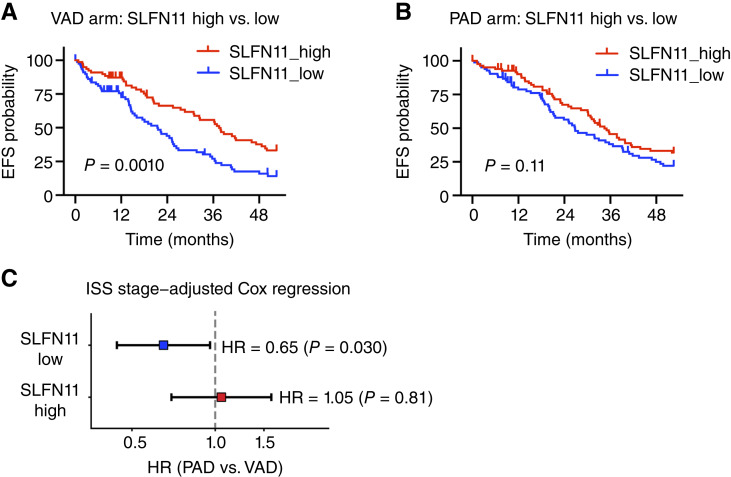
Prediction of the efficacy of BTZ-based therapy based on *SLFN11* expression in the HOVON-65/GMMG-HD4 clinical trial. EFS was analyzed in 327 newly diagnosed patients with multiple myeloma enrolled in the phase III HOVON-65/GMMG-HD4 trial with available pretreatment gene expression data (PAD, *n* = 169; VAD, *n* = 158; refs. 30, 31). Patients were dichotomized into *SLFN11*-high and *SLFN11*-low groups at the cohort median (probe 226743_at, log_2_ = 8.12). **A** and **B,** Kaplan–Meier analyses with unadjusted log-rank tests; (**C**) ISS stage–adjusted Cox regression, excluding patients with unknown ISS stage (*n* = 23). **A,** EFS in the VAD arm (without BTZ). **B,** EFS in the PAD arm (with BTZ). **C,** ISS stage–adjusted hazard ratios (HR) for PAD vs. VAD by *SLFN11* group. Survival data were obtained from the GESTURE repository (48).

In the VAD arm, low *SLFN11* expression appears a significant adverse prognostic factor. *SLFN11*-low patients showed markedly inferior EFS compared with *SLFN11*-high patients (HR = 1.94; 95% CI, 1.31–2.88; log-rank *P* = 0.0010; Fig. 8A). This adverse prognostic effect was abrogated in the PAD arm (log-rank *P* = 0.11; Fig. 8B). Consistent with the prespecified primary analysis of the original HOVON-65/GMMG-HD4 trial (48), which used ISS stage–adjusted Cox regression as its primary statistical approach, we applied the same methodology to this subgroup analysis. ISS stage–adjusted Cox regression (*n* = 305, excluding 23 patients with unknown ISS stage). This analysis demonstrated that PAD significantly improved EFS in *SLFN11*-low patients (HR = 0.65; 95% CI, 0.44–0.96; *P* = 0.030) but not in *SLFN11*-high patients (HR = 1.05; 95% CI, 0.69–1.60; *P* = 0.809; Fig. 8C; Supplementary Fig. S7). Although the formal interaction test did not reach statistical significance (interaction *P* = 0.071), likely reflecting limited statistical power, the directional consistency across all analyses supports the hypothesis that low SLFN11 expression confers selective sensitivity to BTZ-based therapy.

## Discussion

Multiple myeloma remains an incurable malignancy despite advances in proteasome inhibitor–based therapies. Our study provides the first comprehensive characterization of SLFN11 expression in multiple myeloma, proposing a previously unrecognized function of SLFN11 in proteotoxic stress response. We demonstrate that *SLFN11* is consistently highly expressed across multiple myeloma disease stages — intrinsically linked to the plasma cell differentiation program through super-enhancer–mediated transcriptional regulation. We demonstrate that BTZ induces SLFN11 translocation to nucleoli, where it suppresses rRNA synthesis. We also show that SLFN11 KO cells are hypersensitive to BTZ while being, as expected, resistant to TOP1 poisons. This establishes SLFN11 as a dual-function regulator of replication stress and proteotoxic stress responses. These findings reframe SLFN11 not merely as a predictive biomarker for chemotherapies targeting DNA replication but also as an active mediator of proteotoxic stress adaptation in multiple myeloma, with potential relevance to proteasome inhibitor resistance mechanisms.

Multiple myeloma exhibits remarkably high *SLFN11* expression, comparable with or exceeding AML (18), Ewing sarcoma (20), or mesothelioma (19, 49). Notably, this high *SLFN11* expression is maintained throughout malignant transformation and disease progression, suggesting SLFN11’s intrinsic relevance for plasma cell biology. This constitutive expression is coordinated with plasma cell–specific transcriptional programs, as *ZBTB38* and *IRF2*, both identified as essential genes regulated by myeloma-specific super-enhancers (43, 50), showed strong correlation with *SLFN11* expression, most likely reflecting coregulation within this program rather than direct control of *SLFN11* by these factors. Consistently, blocking super-enhancers with JQ1 and THZ1 suppressed *SLFN11* expression (Fig. 3D), providing functional validation of super-enhancer–mediated *SLFN11* regulation in multiple myeloma.

The constitutive SLFN11 expression in multiple myeloma is closely linked to plasma cell identity, as evidenced by its strong correlations with plasma cell differentiation markers, including SDC1, CD38, and TNFRSF17 (Fig. 3A), and its transcriptional regulation by plasma cell–specific super-enhancers (Fig. 3D). Notably, SLFN11 expression varies across multiple myeloma molecular subtypes, with relatively low expression in the CD1 and MF subtypes (Fig. 1D). We speculate that in CCND1-driven multiple myeloma, the balance shifts away from stress-adaptive programs such as SLFN11-mediated translation regulation toward cell-cycle progression.

Plasma cells are characterized by exceptionally high immunoglobulin secretory activity, imposing a substantial burden on the protein quality control machinery. The super-enhancer–mediated upregulation of SLFN11 in this context may therefore reflect an adaptive response to this inherently elevated proteotoxic stress, consistent with the enrichment of ER stress response and UPR pathways in *SLFN11*-high multiple myeloma samples (Fig. 4A). Pathway analyses revealed biological signatures relevant to plasma cell biology. *SLFN11*-high samples show enrichment in ER stress response, UPR, and ERAD pathways critical for managing high secretory burden. Additionally, *SLFN11*-high samples exhibit reduced ribosomal protein gene expression. Together with our functional data showing SLFN11-mediated suppression of rRNA synthesis upon BTZ treatment, this suggests SLFN11 regulates ribosome biogenesis and translation. By tuning down both rRNA and ribosomal protein synthesis, SLFN11 may function as a regulator of translation to limit cellular stress. These functions may be particularly advantageous in plasma cells, which must reconcile high immunoglobulin production with cellular homeostasis.

Our analyses of proliferative activity show that multiple myeloma exhibits relatively low *MKI67* expression compared with other cancer types. Both *MKI67* transcript levels and Ki-67 protein expression (PCPI) increase significantly at relapse (Figs. 1B and 2C), and the preservation of high *SLFN11* expression combined with increased proliferation in recurrent disease is particularly relevant. Both factors are associated with enhanced sensitivity to DNA-damaging agents (see Fig. 7), suggesting that relapsed multiple myeloma may be more susceptible to DNA-damaging therapies, such as TOP1 ADCs (with exatecan and deruxtecan payloads) than newly diagnosed disease.

In untreated conditions, SLFN11 is loosely bound to chromatin (detergent soluble) throughout the nucleus and largely excluded from nucleoli (14, 19, 47, 51). Upon replicative DNA damage by chemotherapeutic agents, SLFN11 forms nuclear foci in replication factories (7, 38, 47, 52). By contrast, BTZ induces SLFN11 translocation to the fibrillar center of nucleoli. This nucleolar binding seems functionally significant. Following BTZ treatment, we find that SLFN11 suppresses rRNA synthesis. The ability of SLFN11 to downregulate rDNA synthesis aligns with a recent publication demonstrating that SLFN11 can bind ribosomal DNA and inhibit rRNA synthesis (9). Our results extend these observations to proteotoxic stress induced by proteasome inhibition, suggesting that SLFN11-mediated rRNA synthesis suppression may represent a conserved mechanism that operates across different types of cellular stresses. Thus, we propose a dual-function model for SLFN11 in multiple myeloma. Under DNA damage stress induced by TOP1 poisons (or chemotherapy-induced replication stress), SLFN11 binds to and blocks replication forks, leading to the death of cells with irreparable damage. In contrast, under proteotoxic stress induced by proteasome inhibition, SLFN11 translocates to nucleoli and suppresses rRNA synthesis, thereby reducing overall protein synthesis (see Supplementary Fig. S6) and mitigating the accumulation of misfolded proteins.

This model has therapeutic implications for multiple myeloma treatment. *SLFN11*-high myeloma cells may possess an intrinsic protective mechanism against proteasome inhibition through SLFN11-mediated control of rRNA synthesis, potentially conferring relative resistance to BTZ. In contrast, *SLFN11*-low myeloma cells lack this protective mechanism and continue rRNA synthesis despite proteasome inhibition, potentially leading to enhanced sensitivity to BTZ. This hypothesis is consistent with our observation that *SLFN11*-low myeloma samples show upregulation of ubiquitin–proteasome system components, suggesting a greater dependency on proteasome function for survival. Based on our model, *SLFN11*-low patients might benefit most from BTZ-based regimens. For patients with very high *SLFN11* expression, combining proteasome inhibitors with agents targeting ribosome biogenesis might further abolish rRNA and protein synthesis. Additionally, given SLFN11’s established role in sensitizing cancer cells to DNA-damaging agents (7, 38), *SLFN11*-high patients with myeloma might benefit from DNA-damaging agents targeting S-phase cells in their treatment regimens. We confirmed this concept functionally by demonstrating that *SLFN11* KO in multiple myeloma cell lines (KMS-27 and KMS-34) conferred resistance to TOP1 poisons (CPT and exatecan), directly validating SLFN11’s role as a determinant of DNA-damaging agent sensitivity in multiple myeloma cells (Fig. 7C and D). This rationale is further strengthened by our observation that proliferative activity (PCPI) increases during disease progression while *SLFN11* expression remains high, potentially enhancing susceptibility to DNA-damaging therapies in the relapsed setting. The strong correlation between *SLFN11* and *BCMA* expression (r = 0.37) provides additional rationale for BCMA-targeted therapies in *SLFN11*-high patients. TOP1 poisons are selectively toxic to replicating cells and are increasingly used as payloads for ADCs (53, 54). Whereas current anti-BCMA ADCs utilize microtubule inhibitor payloads (55, 56), TOP1 payloads such as deruxtecan and exatecan could offer superior efficacy in *SLFN11*-high patients with multiple myeloma, particularly at relapse where both *SLFN11* expression and proliferative activity are high.

Consistent with this mechanistic model, a retrospective subgroup analysis of the HOVON-65/GMMG-HD4 trial provides hypothesis-generating clinical evidence. In the VAD arm (without BTZ), low *SLFN11* expression was a significant adverse prognostic factor (*P* = 0.0010), yet this effect was abrogated in the PAD arm (*P* = 0.11). ISS stage–adjusted Cox regression further demonstrated selective EFS benefit from BTZ specifically in *SLFN11*-low patients (HR = 0.65; *P* = 0.030), supporting the interpretation that *SLFN11*-low multiple myeloma cells, lacking the protective rRNA synthesis suppression mechanism, are selectively sensitized to BTZ-induced proteotoxic stress. Prospective validation in larger cohorts with standardized SLFN11 measurement is required.

Whereas previous studies established the PCPI as a prognostic factor using cross-sectional analyses, our sequential assessment revealed that the PCPI progressively increases with each relapse—a pattern not systematically documented previously (41, 57). Combined with maintained *SLFN11* expression, this suggests a therapeutic window in relapsed disease in which both factors converge to enhance DNA-damaging agent susceptibility.

Several limitations of our study should be acknowledged. Although we demonstrate SLFN11-mediated suppression of rRNA synthesis following BTZ treatment, the detailed molecular mechanisms underlying this function remain to be elucidated. Our mechanistic studies were conducted in cell line models, and confirmation in primary patient samples are needed to strengthen our conclusions, particularly given the difference between high *SLFN11* expression in multiple myeloma cell lines (∼33%) and patient-derived samples (97.2%). Additionally, comprehensive evaluation of SLFN11’s role in multiple myeloma progression and its predictive value for proteasome inhibitor response require further investigations, including prospective clinical studies. *In vivo* studies using SLFN11 KO multiple myeloma models are warranted to further define its role in disease progression.

## Supplementary Material

Supplementary Figure S1 SLFN11 expression in cancer cell lines and multiple myeloma subtypes.

Supplementary Figure S2 SLFN11/CD138 expression patterns and PCPI activity in sequential MM samples.

Supplementary Figure S3 Correlation of SLFN11 expression with lineage markers and transcription factors in multiple myeloma.

Supplementary Figure S4 Heatmap of pathway-related genes and comprehensive GSEA analysis.

Supplementary Figure S5 Bortezomib induces nucleolar recruitment of SLFN11 in multiple myeloma cells and doxycycline-inducible U2OS cells.

Supplementary Figure S6 SLFN11 recruitment to nucleoli limits ribosomal RNA (rRNA) synthesis and affects global translation rates after Bortezomib treatment.

Supplementary Figure S7 Event-free survival by treatment arm stratified by SLFN11 expression in the HOVON-65/GMMG-HD4 trial.

## Data Availability

All datasets analyzed in this study are publicly available: TCGA and MMRF CoMMpass data via the UCSC Xena browser (https://xenabrowser.net); GSE5900, GSE2658, and GSE19784 via the GEO; and HOVON-65/GMMG-HD4 survival data via the GESTURE repository (https://github.com/jubels/GESTURE). Additional raw data are available from the corresponding author upon reasonable request.
