# Supplementary material for: Schlafen 11 Is Overexpressed in Multiple Myeloma and Undergoes Nucleolar Translocation in Response to Bortezomib
Source: Cancer Res Commun. 2026 Jul 27;6(7):1777–93. doi: 10.1158/2767-9764.CRC-26-0162 (PMC13402946; doi:10.1158/2767-9764.CRC-26-0162)
Supplement: Supplementary Figure S1 — SLFN11 expression in cancer cell lines and multiple myeloma subtypes. [file crc-26-0162_supplementary_figure_s1_suppsf1.pdf]

Figure S1.

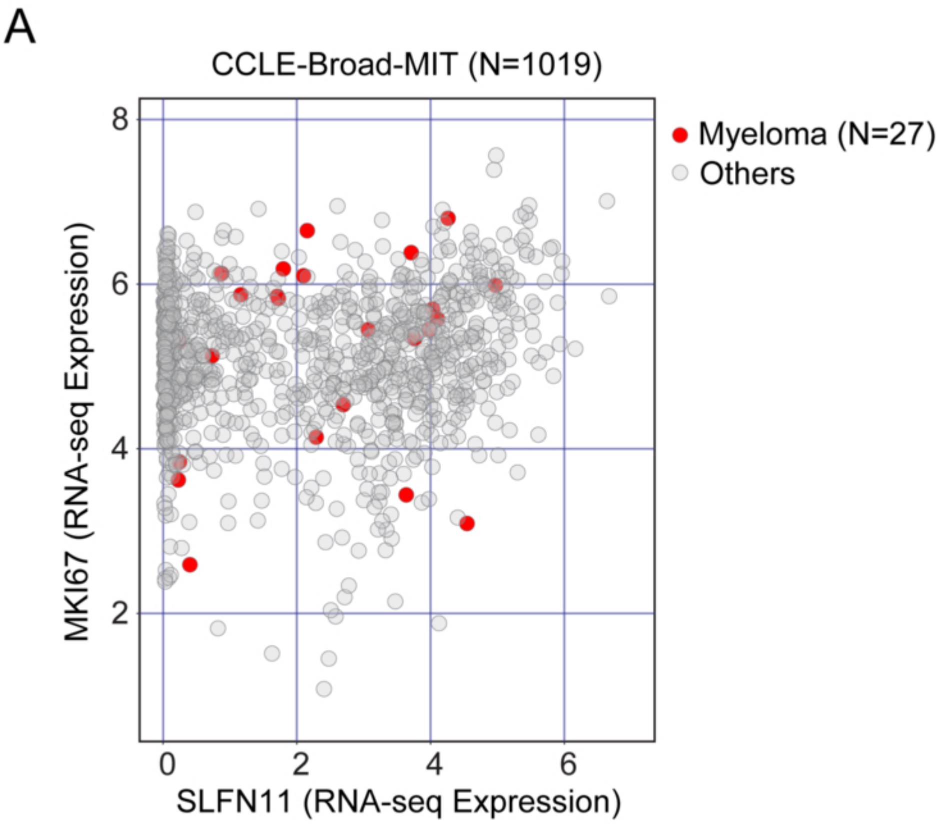
A
CCLE-Broad-MIT (N=1019)
Myeloma (N=27)
Others
MKI67 (RNA-seq Expression)
SLFN11 (RNA-seq Expression)
8
6
4
2
0
2
4
6

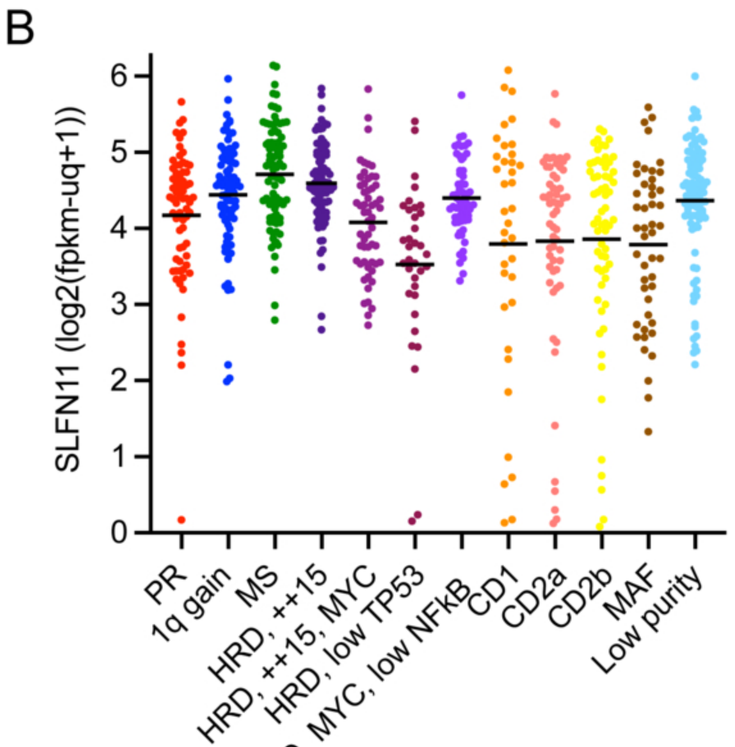
B
SLFN11 (log2(fpkm-uq+1))
6
5
4
3
2
1
0
PR
1q gain
MS
HRD, ++15
HRD, ++15, MYC
HRD, low TP53
HRD, MYC, low NFkB
CD1
CD2a
CD2b
MAF
Low purity

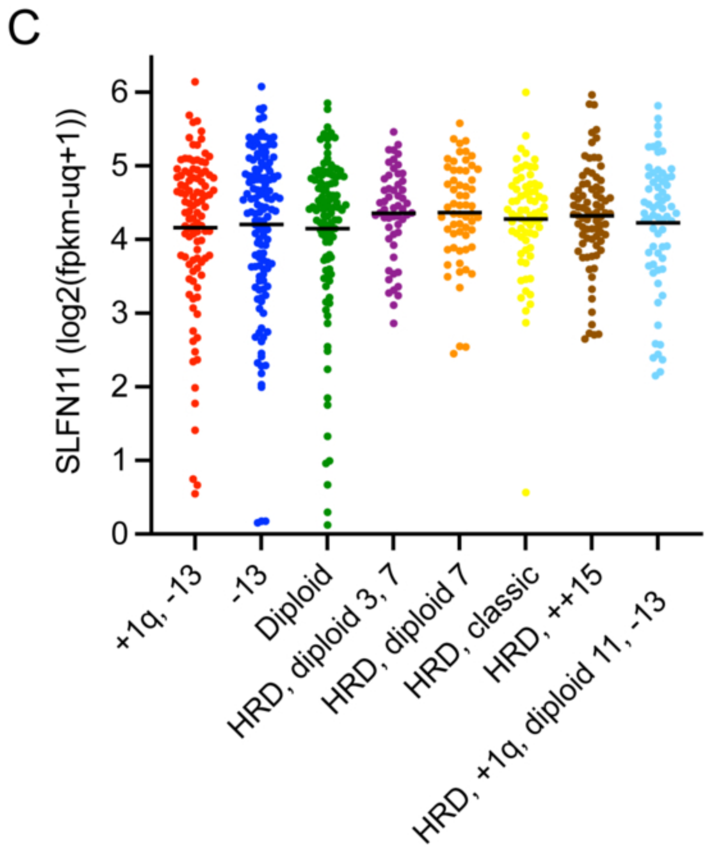
C
SLFN11 (log2(fpkm-uq+1))
6
5
4
3
2
1
0
+1q, -13
-13
Diploid
HRD, diploid 3, 7
HRD, diploid 7
HRD, classic
HRD, ++15
HRD, +1q, diploid 11, -13

Supplementary Figure S1. SLFN11 expression in cancer cell lines and multiple myeloma subtypes. (A) Scatter plot showing the relationship between SLFN11 and MKI67 (RNA-seq expression) across the 1,019 cancer cell lines in the CCLE-Broad-MIT database. Multiple myeloma cell lines (N=27, red) and other cancer cell lines (grey) are shown (data accessed through CellMinerCDB (https://discover.nci.nih.gov/cellminercdb/). (B) SLFN11 expression levels (log2(fpkm uq+1)) across subtypes of multiple myeloma as defined by Maura et al. (Nature Genetics, 2024). Subtypes include PR, 1q gain, MS, HRD with various genomic alterations (++15, MYC, low TP53, low NFκB), CD1, CD2a, CD2b, MAF/MAFB, and low purity samples. Bars represent mean values. (C) SLFN11 expression levels across copy number-based subtypes of multiple myeloma as defined in the same study. Subtypes include +1q/-13, -13, diploid, and HRD with various ploidy patterns. Bars represent mean values.
