# Supplementary material for: Schlafen 11 Is Overexpressed in Multiple Myeloma and Undergoes Nucleolar Translocation in Response to Bortezomib
Source: Cancer Res Commun. 2026 Jul 27;6(7):1777–93. doi: 10.1158/2767-9764.CRC-26-0162 (PMC13402946; doi:10.1158/2767-9764.CRC-26-0162)
Supplement: Supplementary Figure S2 — SLFN11/CD138 expression patterns and PCPI activity in sequential MM samples. [file crc-26-0162_supplementary_figure_s2_suppsf2.pdf]

Figure S2.

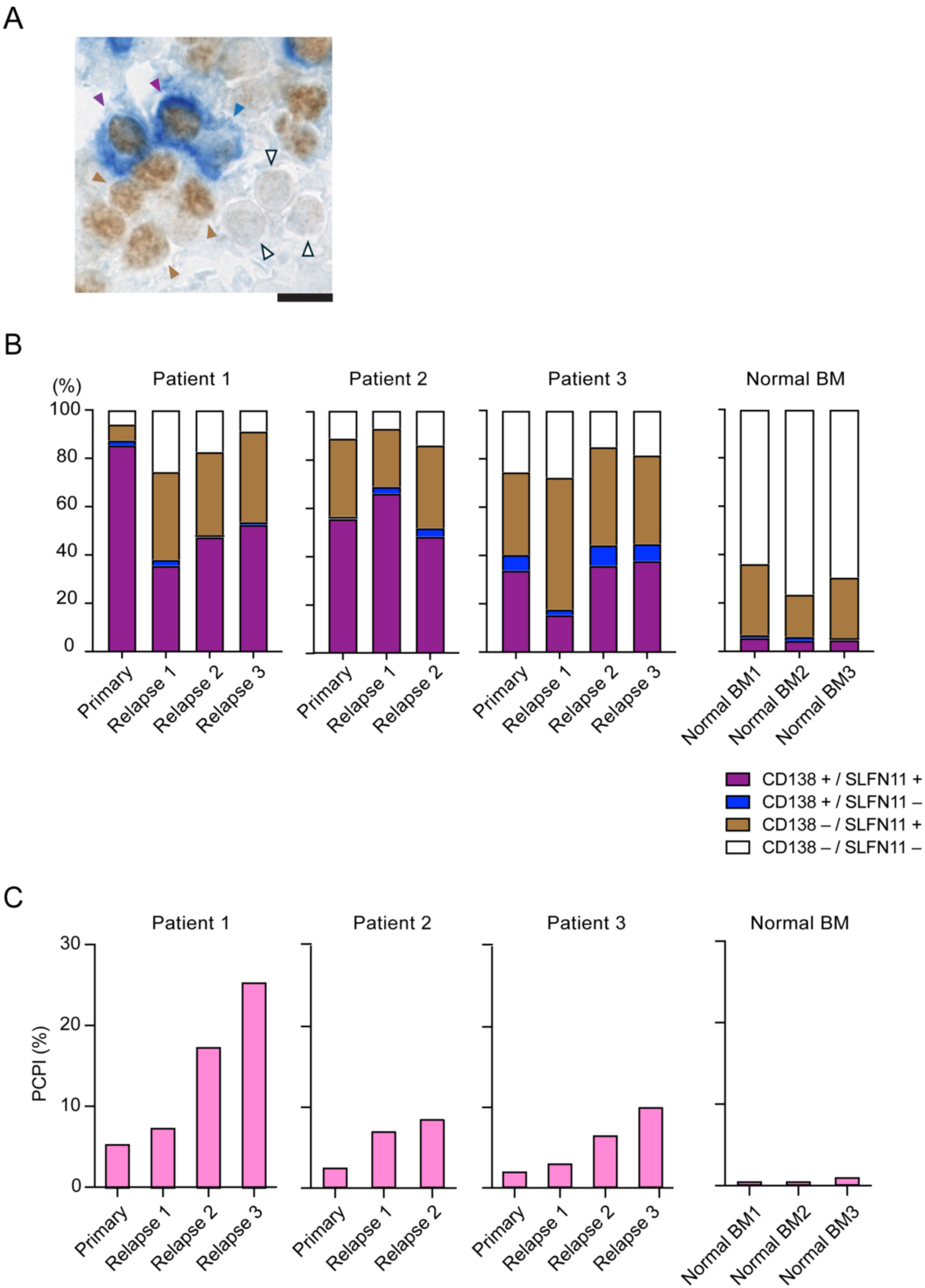
A
B
Patient 1
Patient 2
Patient 3
Normal BM
(%)
100
80
60
40
20
0
Primary
Relapse 1
Relapse 2
Relapse 3
Normal BM1
Normal BM2
Normal BM3
CD138 + / SLFN11 +
CD138 + / SLFN11 –
CD138 – / SLFN11 +
CD138 – / SLFN11 –
C
PCPI (%)
30
20
10
0

Supplementary Figure S2. SLFN11/CD138 expression patterns and PCPI activity in sequential MM samples. (A) High-magnification image of dual immunohistochemical staining for CD138 (blue, membrane) and SLFN11 (brown, nuclear) showing four distinct cell populations. Purple arrowheads indicate CD138+/SLFN11+ cells, blue arrowheads CD138+/SLFN11- cells, brown arrowheads CD138-/SLFN11+ cells, and white arrowheads CD138-/SLFN11- cells. Scale bar: 10 μm. (B) Quantitative analysis of the 4 cell populations in sequential bone marrow samples from three MM patients (Patients 1-3 from Figure 2) at primary diagnosis and through multiple relapses. Normal bone marrow controls are shown for comparison. Cell populations were quantified by counting 1,000 cells per sample (or 500 cells for low-density samples). (C) Plasma Cell Proliferation Index (PCPI) showing progressive increases with each relapse in the 3 patients examined. PCPI was calculated as the percentage of CD138-positive cells co-expressing Ki-67 by counting 200 CD138-positive cells per sample. Normal bone marrow samples show minimal PCPI values.
