# Supplementary material for: Schlafen 11 Is Overexpressed in Multiple Myeloma and Undergoes Nucleolar Translocation in Response to Bortezomib
Source: Cancer Res Commun. 2026 Jul 27;6(7):1777–93. doi: 10.1158/2767-9764.CRC-26-0162 (PMC13402946; doi:10.1158/2767-9764.CRC-26-0162)
Supplement: Supplementary Figure S3 — Correlation of SLFN11 expression with lineage markers and transcription factors in multiple myeloma. [file crc-26-0162_supplementary_figure_s3_suppsf3.pdf]

Figure S3.

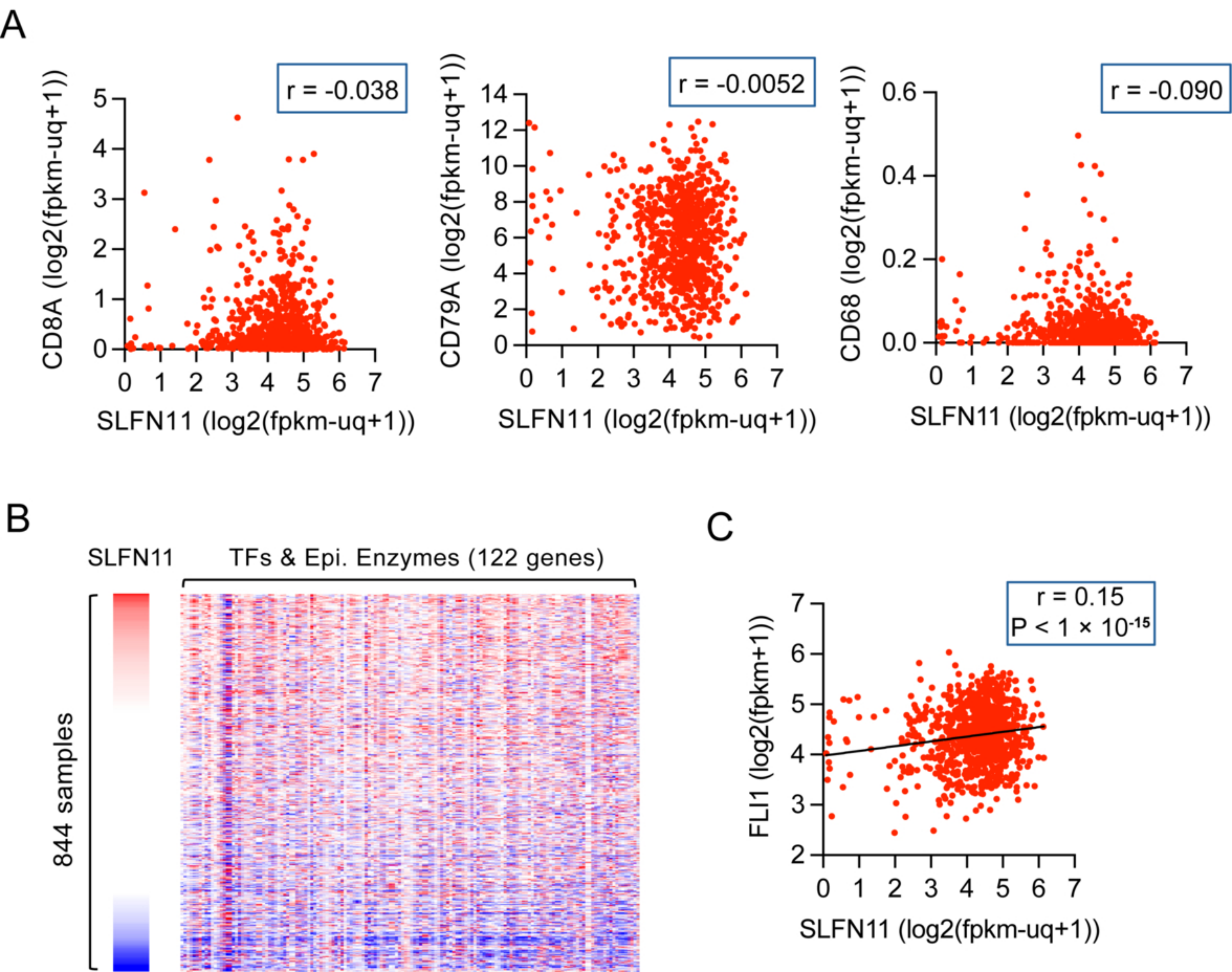
A
r = -0.038
CD8A (log2(fpkm-uq+1))
SLFN11 (log2(fpkm-uq+1))
r = -0.0052
CD79A (log2(fpkm-uq+1))
SLFN11 (log2(fpkm-uq+1))
r = -0.090
CD68 (log2(fpkm-uq+1))
SLFN11 (log2(fpkm-uq+1))
B
SLFN11
TFs & Epi. Enzymes (122 genes)
844 samples
C
r = 0.15
P < 1 × 10-15
FLI1 (log2(fpkm+1))
SLFN11 (log2(fpkm-uq+1))

Supplementary Figure S3. Correlation of SLFN11 expression with lineage markers and transcription factors in multiple myeloma. (A) Scatter plots showing lack of correlation between SLFN11 expression and non-plasma cell lineage markers in multiple myeloma samples (n = 844): CD8A (T-cell marker, r = -0.038), CD79A (B-cell marker, r = -0.0052), and CD68 (monocyte/macrophage marker, r = -0.090), contrasting with the positive correlations observed with plasma cell markers in Figure 3C-E. (B) Heatmap showing correlation between SLFN11 expression and all 122 transcription factors and epigenetic enzymes involved in plasma cell differentiation across 844 multiple myeloma samples, expanding on the top correlations shown in Figure 3D. (C) Scatter plot showing the correlation between SLFN11 and FLI1 (r = 0.15, P < 1 × 10-15), a known transcriptional regulator of SLFN11 expression.
