# Supplementary material for: Schlafen 11 Is Overexpressed in Multiple Myeloma and Undergoes Nucleolar Translocation in Response to Bortezomib
Source: Cancer Res Commun. 2026 Jul 27;6(7):1777–93. doi: 10.1158/2767-9764.CRC-26-0162 (PMC13402946; doi:10.1158/2767-9764.CRC-26-0162)
Supplement: Supplementary Figure S4 — Heatmap of pathway-related genes and comprehensive GSEA analysis. [file crc-26-0162_supplementary_figure_s4_suppsf4.pdf]

Figure S4.

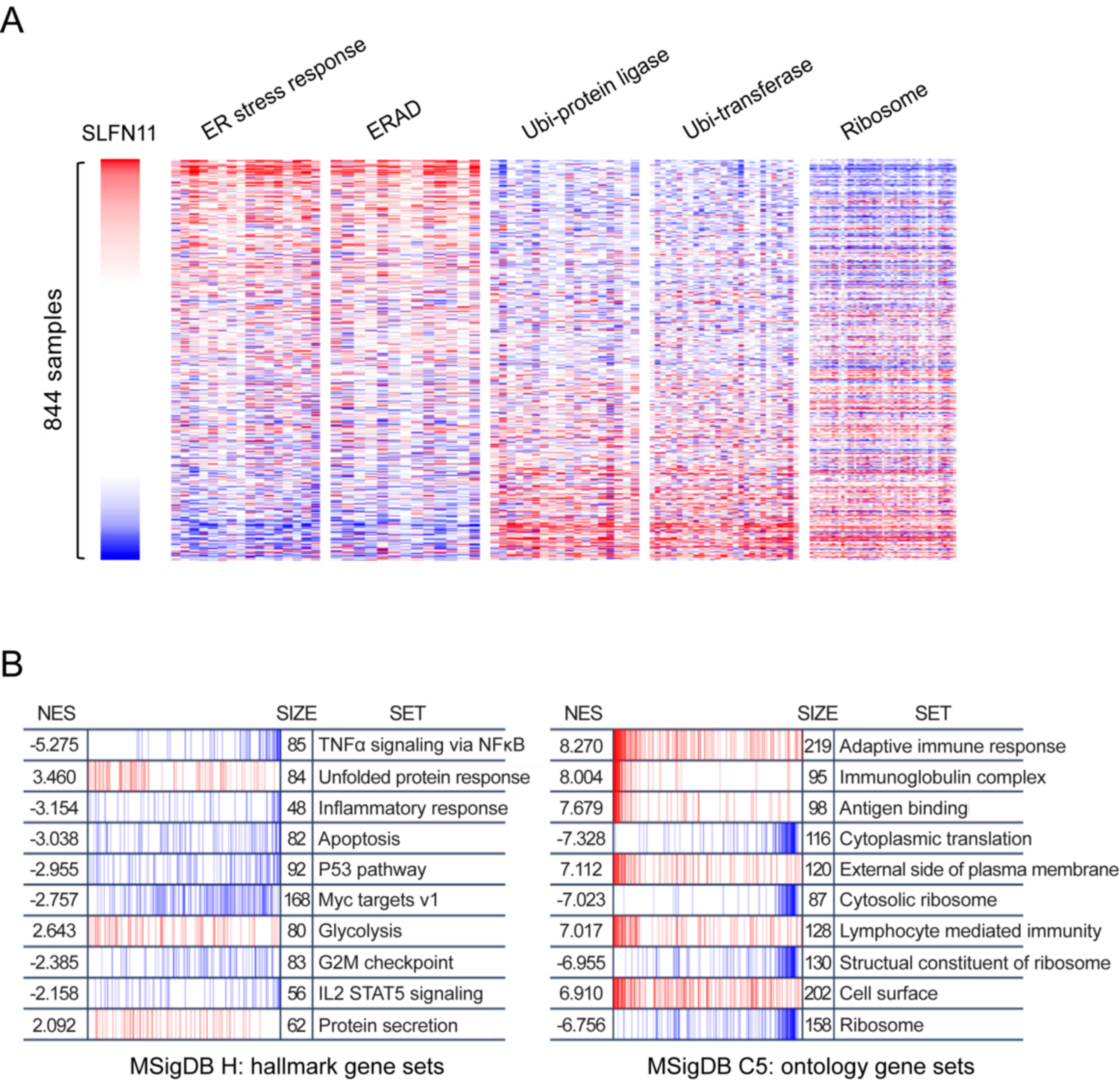
A
SLFN11
ER stress response
ERAD
Ubi-protein ligase
Ubi-transferase
Ribosome
844 samples
B
NES
SIZE
SET
-5.275 85 TNFα signaling via NFκB
3.460 84 Unfolded protein response
-3.154 48 Inflammatory response
-3.038 82 Apoptosis
-2.955 92 P53 pathway
-2.757 168 Myc targets v1
2.643 80 Glycolysis
-2.385 83 G2M checkpoint
-2.158 56 IL2 STAT5 signaling
2.092 62 Protein secretion
MSigDB H: hallmark gene sets
NES
SIZE
SET
8.270 219 Adaptive immune response
8.004 95 Immunoglobulin complex
7.679 98 Antigen binding
-7.328 116 Cytoplasmic translation
7.112 120 External side of plasma membrane
-7.023 87 Cytosolic ribosome
7.017 128 Lymphocyte mediated immunity
-6.955 130 Structual constituent of ribosome
6.910 202 Cell surface
-6.756 158 Ribosome
MSigDB C5: ontology gene sets

Supplementary Figure S4. Heatmap of pathway-related genes and comprehensive GSEA analysis. (A) Heatmap showing expression patterns of differentially expressed genes across 844 multiple myeloma samples ordered by SLFN11 expression (high to low). Genes are grouped by functional pathways: ER stress response, ERAD (ER-associated degradation), ubiquitin-protein ligase, ubiquitin-transferase, and ribosome. Red indicates high expression; blue indicates low expression. (B) Comprehensive Gene Set Enrichment Analysis (GSEA) results comparing SLFN11-high versus SLFN11-low multiple myeloma samples (n = 844, divided by median expression). Left panel: MSigDB Hallmark gene sets. Right panel: MSigDB C5 ontology gene sets. Red indicates positive enrichment in SLFN11-high samples; blue indicates negative enrichment (positive in SLFN11-low samples). Selected representative enrichment plots are shown in Figure 4B. NES: Normalized Enrichment Score; SIZE: gene set size.
