# Supplementary material for: Schlafen 11 Is Overexpressed in Multiple Myeloma and Undergoes Nucleolar Translocation in Response to Bortezomib
Source: Cancer Res Commun. 2026 Jul 27;6(7):1777–93. doi: 10.1158/2767-9764.CRC-26-0162 (PMC13402946; doi:10.1158/2767-9764.CRC-26-0162)
Supplement: Supplementary Figure S5 — Bortezomib induces nucleolar recruitment of SLFN11 in multiple myeloma cells and doxycycline-inducible U2OS cells. [file crc-26-0162_supplementary_figure_s5_suppsf5.pdf]

Figure S5.

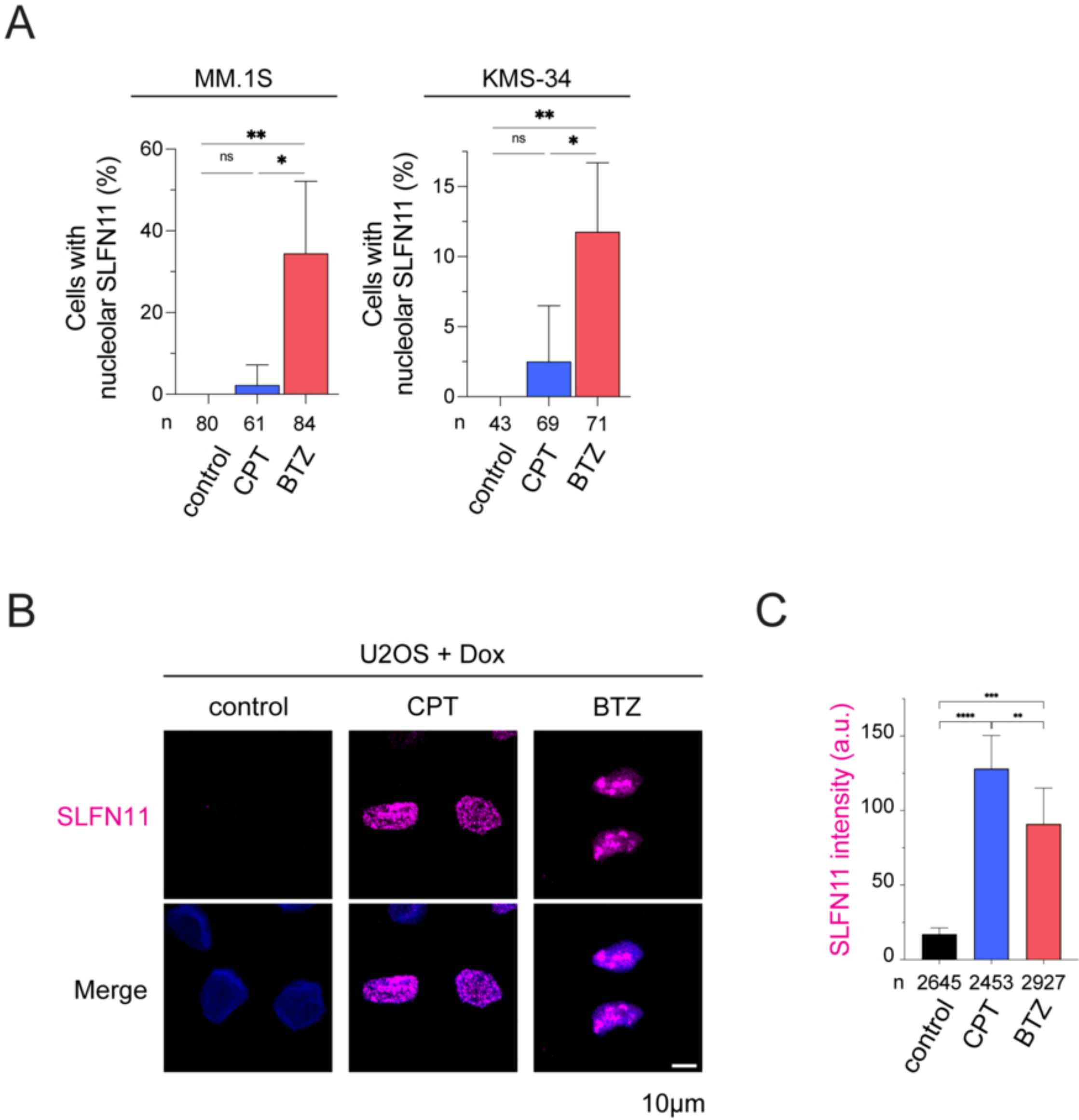

A
MM.1S
KMS-34
Cells with nucleolar SLFN11 (%)
60
40
20
0
15
10
5
**
ns
*
n
80
61
84
43
69
71
control
CPT
BTZ
B
U2OS + Dox
control
CPT
BTZ
SLFN11
Merge
10μm
C
SLFN11 intensity (a.u.)
150
100
50
***
****
n
2645
2453
2927

Supplementary Figure S5. Bortezomib induces nucleolar recruitment of SLFN11 in multiple myeloma cells and doxycycline-inducible U2OS cells. (A) Quantification of the proportion of cells with chromatin-bound SLFN11 foci overlapping with nucleolin in MM.1S and KMS-34 cells (Figure 5D). Cells were treated with camptothecin (CPT) or bortezomib (BTZ) for 4 hours (n = 43–84 cells per condition). Mean ± SD are shown. ns, not significant; *$P < 0.05$, **$P < 0.01$ (one-way ANOVA). (B) Representative immunofluorescence images showing chromatin-bound SLFN11 (magenta, upper panels) and merged images with DAPI (blue, lower panels) in doxycycline-induced U2OS cells. Cells were treated with camptothecin (CPT, positive control for chromatin-bound SLFN11) or bortezomib (BTZ, 1 μM) for 4 hours, followed by pre-extraction, fixation, and staining. CPT induces SLFN11 localization at replication forks, whereas BTZ induces SLFN11 translocation to nucleoli. Scale bar: 10 μm. (C) Quantification of SLFN11 signal intensities in individual U2OS cells (n = 2453–2927 cells per condition). Mean ± SD are shown. **$P < 0.01$, ***$P < 0.001$, ****$P < 0.0001$ (one-way ANOVA). a.u., arbitrary units.
