# Supplementary material for: Schlafen 11 Is Overexpressed in Multiple Myeloma and Undergoes Nucleolar Translocation in Response to Bortezomib
Source: Cancer Res Commun. 2026 Jul 27;6(7):1777–93. doi: 10.1158/2767-9764.CRC-26-0162 (PMC13402946; doi:10.1158/2767-9764.CRC-26-0162)
Supplement: Supplementary Figure S6 — SLFN11 recruitment to nucleoli limits ribosomal RNA (rRNA) synthesis and affects global translation rates after Bortezomib treatment. [file crc-26-0162_supplementary_figure_s6_suppsf6.pdf]

Figure S6.

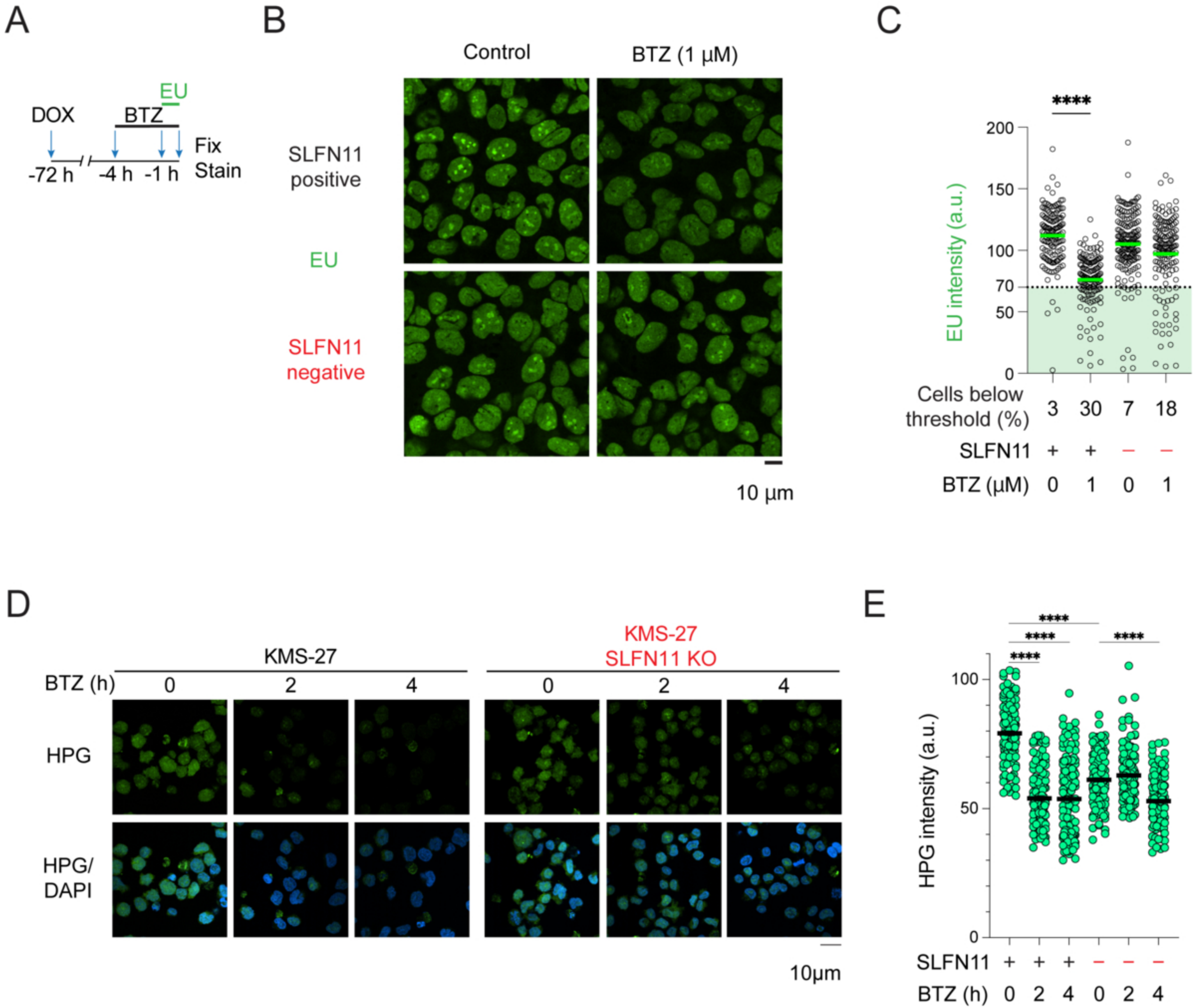
A
EU
DOX
BTZ
Fix
Stain
-72 h
-4 h
-1 h
B
Control
BTZ (1 µM)
SLFN11 positive
EU
SLFN11 negative
10 µm
C
****
EU intensity (a.u.)
200
150
100
70
50
0
Cells below threshold (%)
3
30
7
18
SLFN11
+
+
–
–
BTZ (µM)
0
1
0
1
D
KMS-27
KMS-27 SLFN11 KO
BTZ (h)
0
2
4
0
2
4
HPG
HPG/ DAPI
10µm
E
****
****
****
****
HPG intensity (a.u.)
100
50
0
SLFN11
+
+
+
–
–
–
BTZ (h)
0
2
4
0
2
4

Supplementary Figure S6. SLFN11 recruitment to nucleoli limits ribosomal RNA (rRNA) synthesis and affects global translation rates after Bortezomib treatment. (A) Treatment protocol. Doxycycline (DOX)-inducible SLFN11-expressing U2OS cells were treated with bortezomib (BTZ, 1 μM) for 4 hours. (B) Representative immunofluorescence images showing 5-ethynyluridine (EU) incorporation (green). Scale bar: 10 μm. (C) Quantification of EU signals in individual cells for the indicated treatments (n = 135 – 167 cells per condition). Mean ± SEM are shown. ****P < 0.0001 (one-way ANOVA). a.u., arbitrary units. The threshold for EU (70) was determined based on distributions observed in the control experiment. a.u., arbitrary units. (D) Representative immunofluorescence images showing HPG incorporation (green). WT and SLFN11 KO clones of KMS-27 cells were treated with BTZ (0.1 μM) for indicated timepoints. Scale bar: 10 μm. (E) Quantification of HPG signals in individual cells for the indicated treatments (n = 120 – 159 cells per condition). Mean ± SEM are shown. ****P < 0.0001 (one-way ANOVA). a.u., arbitrary units.
