# Supplementary material for: Schlafen 11 Is Overexpressed in Multiple Myeloma and Undergoes Nucleolar Translocation in Response to Bortezomib
Source: Cancer Res Commun. 2026 Jul 27;6(7):1777–93. doi: 10.1158/2767-9764.CRC-26-0162 (PMC13402946; doi:10.1158/2767-9764.CRC-26-0162)
Supplement: Supplementary Figure S7 — Event-free survival by treatment arm stratified by SLFN11 expression in the HOVON-65/GMMG-HD4 trial. [file crc-26-0162_supplementary_figure_s7_suppsf7.pdf]

Figure S7.

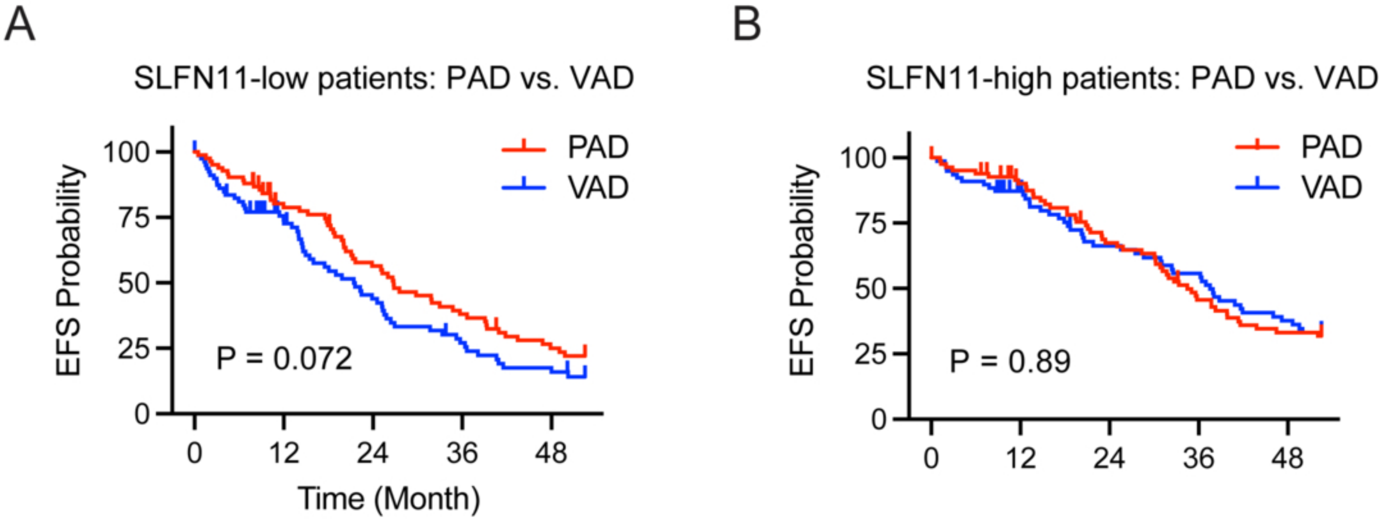
A
SLFN11-low patients: PAD vs. VAD
PAD
VAD
EFS Probability
100
75
50
25
0
P = 0.072
0
12
24
36
48
Time (Month)
B
SLFN11-high patients: PAD vs. VAD
PAD
VAD
EFS Probability
100
75
50
25
0
P = 0.89
0
12
24
36
48

Supplementary Figure S7. Event-free survival by treatment arm stratified by SLFN11 expression in the HOVON-65/GMMG-HD4 trial. Kaplan-Meier analyses comparing PAD versus VAD within each SLFN11 expression subgroup (unadjusted log-rank test).
(A) SLFN11-low patients (n=163; PAD, n=84; VAD, n=79; log-rank P=0.072). (B) SLFN11-high patients (n=164; PAD, n=85; VAD, n=79; log-rank P=0.89). ISS stage-adjusted analyses incorporating both subgroups are presented in Figure 8C. EFS, event-free survival; PAD, bortezomib/doxorubicin/dexamethasone; VAD, vincristine/doxorubicin/dexamethasone.
